# Preventing Benzoquinone‐Based Catalyst Aggregation Enables the One‐Step Synthesis of Highly Conductive Poly(benzodifurandione) without Post‐Reaction Purification

**DOI:** 10.1002/adma.202502426

**Published:** 2025-03-18

**Authors:** Jun‐Da Huang, Qifan Li, Qingqing Wang, Tiefeng Liu, Sang Young Jeong, Sri Harish Kumar Paleti, Tom P. A. van der Pol, Kai Xu, Han‐Yan Wu, Natalie Pinchin, Marc‐Antoine Stoeckel, Wenlong Jin, Aleksandr Perevedentsev, Xianjie Liu, Juan Sebastián Reparaz, Mariano Campoy‐Quiles, Han Young Woo, Christian Müller, Mats Fahlman, Chi‐Yuan Yang, Simone Fabiano

**Affiliations:** ^1^ Laboratory of Organic Electronics Department of Science and Technology Linköping University Norrköping SE‐60174 Sweden; ^2^ Wallenberg Wood Science Center Department of Science and Technology Linköping University Norrköping SE‐60174 Sweden; ^3^ n‐Ink AB Källvindsgatan 5 Norrköping SE‐60240 Sweden; ^4^ Wallenberg Initiative Materials Science for Sustainability Department of Science and Technology Linköping University Norrköping SE‐60174 Sweden; ^5^ Department of Chemistry College of Science Korea University Seoul 136‐713 Republic of Korea; ^6^ Department of Chemistry and Chemical Engineering Chalmers University of Technology Göteborg 41296 Sweden; ^7^ Department of Nanostructured Materials Institut de Ciència de Materials de Barcelona ICMAB‐CSIC Bellaterra E‐08193 Spain; ^8^ Wallenberg Wood Science Center Chalmers University of Technology Göteborg 41296 Sweden; ^9^ Stellenbosch Institute for Advanced Study Wallenberg Research Centre at Stellenbosch University Stellenbosch 7600 South Africa

**Keywords:** catalyst aggregation, n‐type conductive polymers, PBFDO, scalable synthesis, thermoelectric properties

## Abstract

Conductive polymers have become crucial in advancing various electronic applications. While p‐type materials like poly(3,4‐ethylenedioxythiophene):polystyrene sulfonate (PEDOT:PSS) are widely used and produced at scale, the development of high‐performance n‐type polymers has lagged due to challenges in synthesis and scalability. In this work, a novel method is introduced to synthesize the highly conductive n‐type polymer poly(benzodifurandione) (PBFDO) using α‐tocopherylquinone (α‐TQ) as a catalyst. This approach eliminates the need for post‐reaction dialysis, a major obstacle to large‐scale PBFDO production. By preventing catalyst aggregation, high electrical conductivity (>1320 S cm^−1^) is achieved, which remains stable in air for over 180 d, significantly simplifying the process. The α‐TQ‐synthesized PBFDO also exhibits excellent thermoelectric properties, with a power factor exceeding 100 µW m^−1^ K^−2^, placing it among the highest‐performing n‐type thermoelectric polymers. Additionally, residual α‐TQ acts as a plasticizer, reducing the elastic modulus by over tenfold while maintaining high conductivity, making this material suitable for mechanically compliant electronics. Similarly, residual α‐TQ lowers the thermal conductivity of PBFDO by more than an order of magnitude. The process is scalable, as demonstrated by producing high‐conductivity ink in a 20 L reactor. This work presents an efficient and sustainable approach for large‐scale n‐type polymer production.

## Graphical abstract

α‐Tocopherylquinone (α‐TQ), the oxidation product of α‐tocopherol (α‐TOH), catalyzes the oxidative polymerization of 3,7‐dihydrobenzo[1,2‐b:4,5‐b′]difuran‐2,6‐dione (HBFDO). The saturated aliphatic chiral side chain of α‐TQ suppresses its crystallization, enabling high‐conductivity polymer ink without needing post‐polymerization dialysis. This scalable method, tested in a 20 L reactor, yields ready‐to‐use poly(benzodifurandione) (PBFDO) ink.

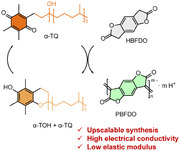

## 1 Introduction

Conductive polymers combine the high mechanical flexibility and cost‐effective solution processability of traditional polymers with the electrical properties of (semi‐)conductors.^[^ ^1^, ^2^, ^3^ ^]^ These distinctive characteristics make them highly promising for a broad range of applications, including energy harvesting and storage devices,^[^ ^4^, ^5^, ^6^ ^]^ wearable sensors,^[^ ^7^, ^8^, ^9^ ^]^ bioelectronics,^[^ ^10^, ^11^, ^12^, ^13^ ^]^ flexible displays,^[^ ^14^ ^]^ and more. Among these, the hole‐transporting (p‐type) material poly(3,4‐ethylenedioxythiophene):polystyrene sulfonate (PEDOT:PSS) is particularly noteworthy, with production reaching multiton scales annually.^[^ ^15^ ^]^ Due to its easy solution processability in water, commercial availability, and tunable electrical conductivity across several orders of magnitude, PEDOT:PSS is widely used in various applications, such as organic solar cells and light‐emitting diodes,^[^ ^16^, ^17^ ^]^ electrochromic displays,^[^ ^18^ ^]^ actuators,^[^ ^19^ ^]^ water purifiers,^[^ ^20^ ^]^ drug delivery systems,^[^ ^21^, ^22^ ^]^ electrochemical transistors,^[^ ^23^, ^24^, ^25^ ^]^ sensors,^[^ ^26^ ^]^ batteries^[^ ^27^, ^28^ ^]^ and supercapacitors,^[^ ^29^ ^]^ stretchable and wearable devices,^[^ ^30^, ^31^ ^]^ thermoelectric generators,^[^ ^32^, ^33^ ^]^ and neuromorphic hardware.^[^ ^34^ ^]^ However, progress in optoelectronics, bioelectronics, and energy applications often depends on the synergistic integration of both high‐performance p‐type and n‐type (electron‐transporting) materials, with the latter lagging behind until recent developments.^[^ ^35^, ^36^ ^]^

While significant progress has been made in developing high‐performance n‐type conductive polymers in recent years, the complexity of their monomer synthesis—typically involving an amide functionalized TBDPPV monomer,^[^ ^37^ ^]^ a boron–nitrogen coordination‐bond‐based PBN‐19 monomer,^[^ ^38^ ^]^ or a bithiophene imide‐based monomer CNI‐2Br^[^ ^39^ ^]^—and relatively low yields constrain their scalability for commercial and industrial applications.^[^ ^40^, ^41^ ^]^ Additionally, these polymers typically exhibit much lower conductivity (< 100 S cm^−1^)^[^ ^37^, ^39^, ^42^, ^43^, ^44^ ^]^ compared to p‐type materials like PEDOT:PSS (> 1000 S cm^−1^).^[^ ^45^, ^46^, ^47^ ^]^ Recently, Tang et al. reported the benchmark n‐type polymer poly(benzodifurandione) (PBFDO), which achieved the highest electrical conductivity of any n‐type conductive polymer to date, exceeding 1000 S cm^−1^.^[^ ^48^ ^]^ The high electrical conductivity of PBFDO has already enabled the development of various high‐performance electronic devices, including organic electrochemical transistors,^[^ ^49^, ^50^, ^51^ ^]^ electrochromic displays,^[^ ^52^ ^]^ transparent electrodes,^[^ ^53^ ^]^ and thermoelectric textiles.^[^ ^33^, ^54^, ^55^ ^]^

For the synthesis of PBFDO, tetramethylquinone (TMQ) is typically used as the catalyst for the oxidative polymerization of 3,7‐dihydrobenzo[1,2‐b:4,5‐b′]difuran‐2,6‐dione (HBFDO). However, due to the highly crystalline nature of TMQ and its reduced form, tetramethylhydroquinone (TMQH), the purification steps of PBFDO require dialysis to remove the catalyst after the polymerization to ensure maximum electrical conductivity. This dialysis process, however, requires large volumes of dimethyl sulfoxide (DMSO)—a solvent that carries some health risks^[^ ^56^ ^]^—and takes considerable time (typically several weeks), making it unsuitable for efficient large‐batch production. Additionally, the high costs associated with both the consumption and disposal of DMSO indicate that TMQ is not the optimal catalyst for the commercial production of PBFDO. Mei and co‐workers reported the synthesis of PBFDO using copper acetate^[^ ^52^, ^57^ ^]^ or selenium dioxide^[^ ^58^ ^]^ as catalysts. However, both methods still require post‐treatment processes such as dialysis or centrifugation plus filtration, which may impact production costs for industrial applications. Recently, we demonstrated that using 3‐(2,4,5‐trimethyl‐3,6‐dioxocyclohexa‐1,4‐dien‐1‐yl)propanoic acid (TMQ‐PA) as a catalyst enables the synthesis of PBFDO derivatives in water.^[^ ^32^ ^]^ Upon reaction with HBFDO, TMQ‐PA converts to 6‐hydroxy‐5,7,8‐trimethylchroman‐2‐one (HTMCO), which is water‐insoluble and can be extracted using diethyl ether. However, the reaction requires a base environment, which opens some of PBFDO's monomer units, thereby reducing its overall electrical conductivity to around 60 S cm^−1^. While these developments represent a significant step toward developing highly conductive n‐type polymers, they also underscore the need for more efficient and safer alternative approaches.

Here, we demonstrate that preventing the aggregation of benzoquinone‐based catalysts eliminates the need for post‐reaction dialysis in PBFDO synthesis. We utilize α‐tocopherylquinone (α‐TQ), the oxidation product of rac‐α‐tocopherol (α‐TOH, one of the eight isoforms of vitamin E^[^ ^59^ ^]^), to catalyze the one‐step oxidative polymerization of HBFDO in DMSO. Unlike TMQ, α‐TQ features a saturated aliphatic chiral side chain tethered to the 3,5,6‐trimethyl‐1,4‐benzoquinone moiety, which prevents crystallization upon reduction to α‐TOH,^[^ ^60^, ^61^ ^]^ allowing the catalyst to remain in solution post‐polymerization. This results in a polymer ink that forms highly conductive films independent of dialysis (1321 ± 67 S cm^−1^ before and 1333 ± 60 S cm^−1^ after dialysis). These values more than double those of TMQ‐synthesized PBFDO before dialysis (508 ± 42 S cm^−1^) and are comparable to the dialyzed TMQ‐synthesized PBFDO films (1351 ± 85 S cm^−1^). Moreover, the Seebeck coefficient of α‐TQ‐synthesized PBFDO remains consistent at approximately ‐30.9 µV K^−1^ before and after dialysis, yielding a power factor exceeding 100 µW m^−1^ K^−2^—surpassing TMQ‐synthesized PBFDO and ranking among the highest reported for n‐type polymers. When integrated into flexible thermocouples with PEDOT:PSS as the p‐type leg, power outputs reached up to 26 nW at Δ*T* = 50 K. Additionally, α‐TQ/α‐TOH residues act as plasticizers, reducing the elastic modulus of α‐TQ‐synthesized PBFDO by over tenfold compared to TMQ‐synthesized PBFDO (from 2.4 to 0.12 GPa), while preserving high electrical conductivity and excellent thermal stability. A similar trend is observed in thermal conductivity, with α‐TQ‐synthesized PBFDO exhibiting a thermal conductivity more than an order of magnitude lower than that of its TMQ‐synthesized counterpart (0.48 W m^−1^ K^−1^ vs 6.04 W m^−1^ K^−1^). The scalability of this method is further highlighted by successful upscaling in a 20 L reactor, producing a high‐conductivity PBFDO ink ready for use without further purification.

## 2 Results and Discussion

### 2.1 Catalyst Design, Polymer Synthesis, and Characterization

**Figure 1:**
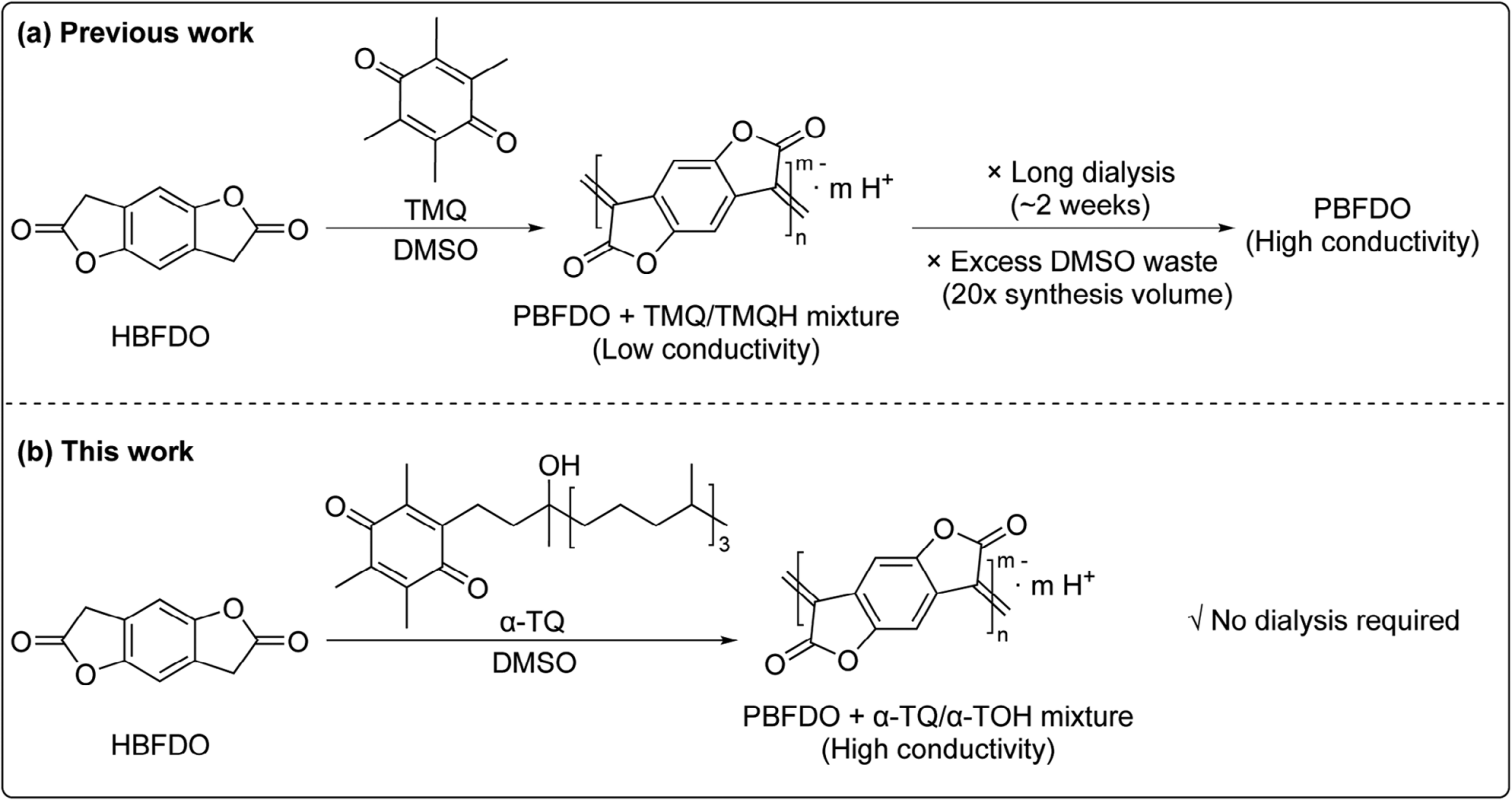
a) Schematic diagram of PBFDO synthesis using TMQ, followed by post‐synthesis dialysis treatment. b) Schematic diagram of PBFDO synthesis using α‐TQ.

The chemical structure of α‐TOH is shown in Figure S1 in the Supporting Information. It is characterized by a chromanol ring with a hydroxyl group at position 6 and a long phytyl chain. α‐TOH can be oxidized to α‐TQ in the presence of ferric trichloride (FeCl_3_) at yields >90% (Figure S1b, Supporting Information).^[^ ^62^ ^]^ The presence of chiral centers in the phytyl chain of α‐TQ and α‐TOH plays a crucial role in suppressing aggregation, promoting a more stable oily form compared to TMQ (Figure S2, Supporting Information). The quinone ring in α‐TQ is highly reactive and can oxidize HBFDO in DMSO to form PBFDO through a polymerization mechanism similar to that reported for TMQ as the catalyst (**Figure** **1**).^[^ ^48^ ^]^ Upon reaction, α‐TQ is reduced to α‐TOH, which can be either re‐oxidized to α‐TQ after extraction with diethyl ether (Figure S3, Supporting Information) or left in the PBFDO solution without further purification (vide infra). The calculated E‐factor^[^ ^63^ ^]^ of α‐TQ‐synthesized PBFDO (without dialysis) is the smallest among all reported n‐type polymers (Figure S4, Supporting Information).

**Figure 2:**
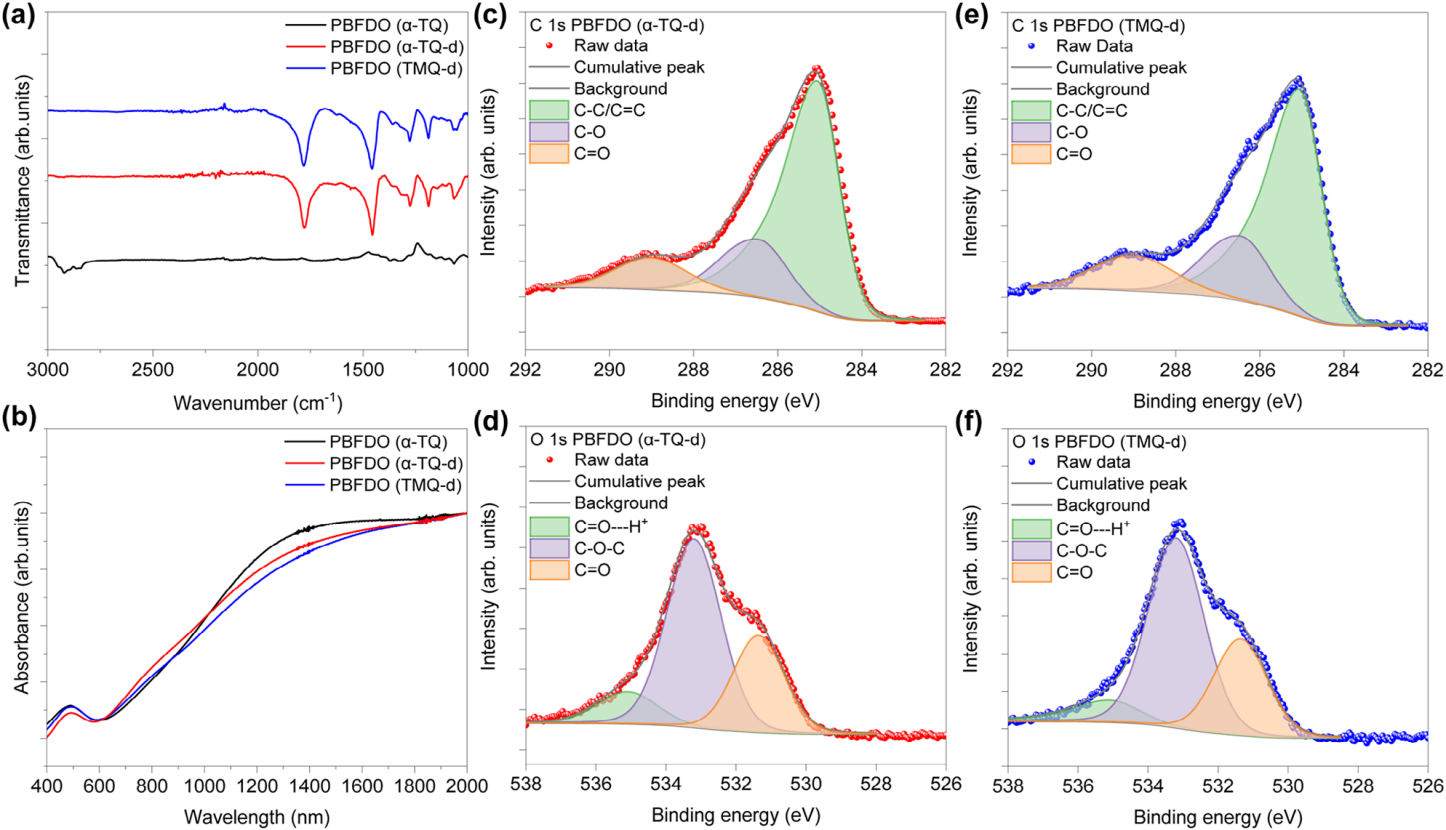
a) FTIR spectra of α‐TQ‐synthesized PBFDO (before and after dialysis) and TMQ‐synthesized PBFDO (after dialysis). b) UV–vis spectra of α‐TQ‐synthesized PBFDO (before and after dialysis) and TMQ‐synthesized PBFDO (after dialysis). c) XPS C(1s) and d) O(1s) spectra of α‐TQ‐synthesized PBFDO (after dialysis). e) XPS C(1s) and f) O(1s) spectra of TMQ‐synthesized PBFDO (after dialysis).

Fourier‐transform infrared spectroscopy (FTIR) was performed to investigate the chemical structure of PBFDO synthesized using α‐TQ and to compare it with PBFDO synthesized using TMQ as the catalyst (**Figure** **2**a). The FTIR spectrum of α‐TQ‐synthesized PBFDO before dialysis [PBFDO (α‐TQ)] shows a pronounced peak at 3000–2750 cm^−1^, assigned to alkane C─H, but lacks the characteristic C═O peak at 1780 cm^−1^ and the fingerprint region below 2000 cm^−1^ (black line). We attributed this to residual α‐TQ/α‐TOH in the unpurified mixtures, which interact with the PBFDO backbone, masking its characteristic features (see further details in Figure S5, Supporting Information). After dialysis [PBFDO (α‐TQ‐d)], the FTIR spectrum of α‐TQ‐synthesized PBFDO (red line) shows a strong C═O peak at 1781 cm^−1^ and a fingerprint region identical to that of dialyzed PBFDO synthesized using TMQ [PBFDO (TMQ‐d)] (blue line).^[^ ^32^ ^]^ The ultraviolet–visible‐near‐infrared (UV‐vis‐NIR) absorption spectra of α‐TQ‐synthesized PBFDO, before and after dialysis, show a pronounced absorption band extending beyond 2000 nm, attributed to the presence of polaron/bipolaron species, which is qualitatively similar to that observed for TMQ‐synthesized PBFDO (Figure 2b).^[^ ^48^ ^]^

X‐ray photoelectron spectroscopy (XPS) was conducted to further characterize the chemical structure of PBFDO synthesized from α‐TQ (Figure 2c–f). Note that only dialyzed samples were considered for these measurements to exclude the impact of catalyst residues and ensure an accurate interpretation of the spectra. Analysis of the XPS C(1s) and O(1s) spectra of α‐TQ‐synthesized PBFDO reveals three components for the C(1s) peaks located at 285.1 eV (C─C/C═C), 286.6 eV (C─O), and 289.1 eV (C═O) (Figure 2c), and three components for the O(1s) peaks located at 535.1 eV (C═O─H^+^), 531.4 eV (C═O), and 533.2 eV (C─O─C) (Figure 2d). The shapes and binding energies of these peaks are comparable to those of PBFDO synthesized using TMQ (Figure 2e,f).

### 2.2 Film Microstructure Characterization

**Figure 3:**
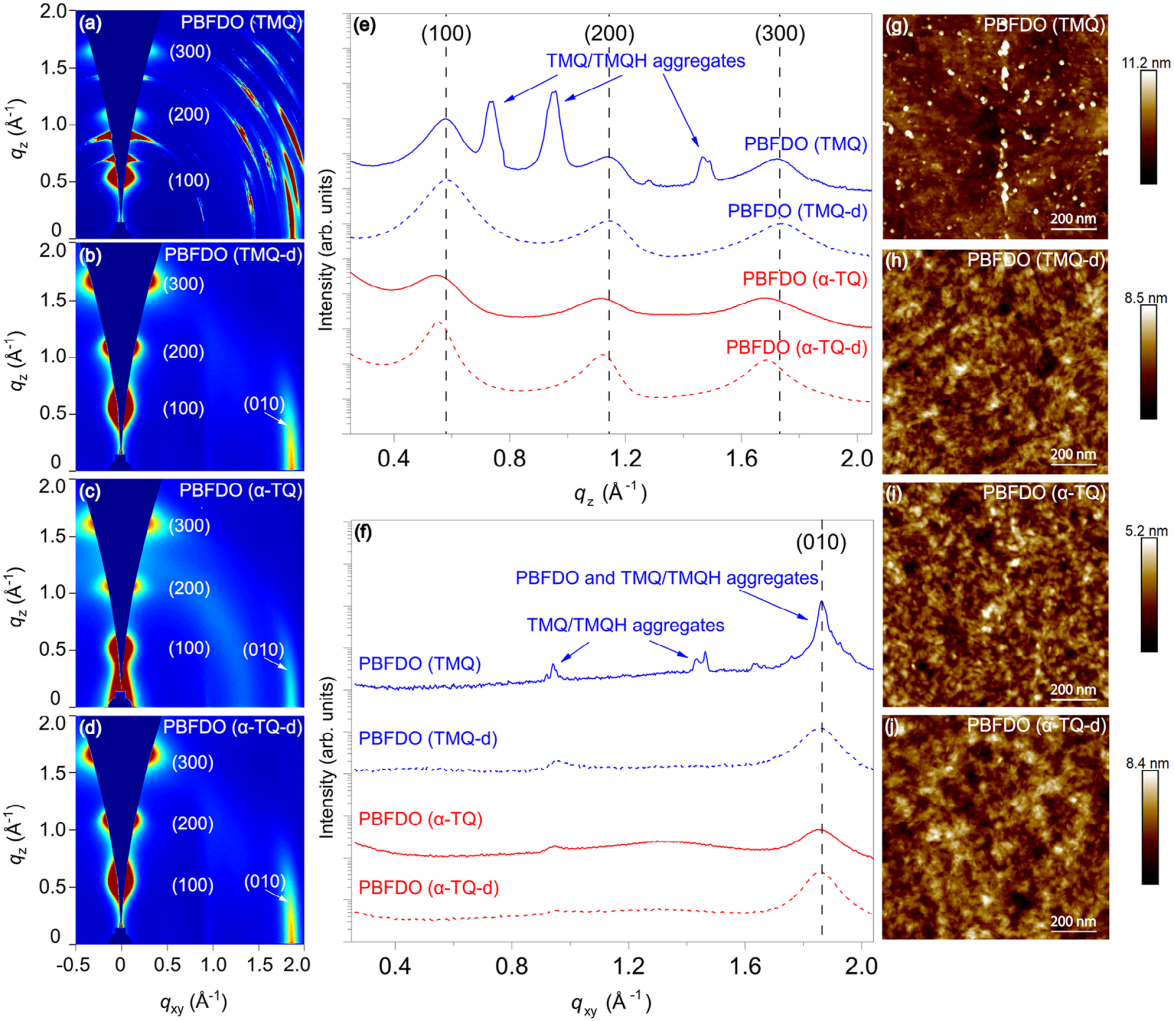
a–d) 2D GIWAXS patterns of a) TMQ‐synthesized PBFDO (before dialysis), b) TMQ‐synthesized PBFDO (after dialysis), c) α‐TQ‐synthesized PBFDO (before dialysis), and d) α‐TQ‐synthesized PBFDO (after dialysis) thin films. Corresponding 1D line cuts in the e) out‐of‐plane and f) in‐plane direction. g–j) AFM height images of g) TMQ‐synthesized PBFDO (before dialysis), h) TMQ‐synthesized PBFDO (after dialysis), i) α‐TQ‐synthesized PBFDO (before dialysis), and j) α‐TQ‐synthesized PBFDO (after dialysis) thin films.

Next, we investigated the microstructure of PBFDO thin films using grazing‐incidence wide‐angle X‐ray scattering (GIWAXS) (**Figure** **3**a–f). The 2D GIWAXS images of TMQ‐synthesized PBFDO (before and after dialysis) and α‐TQ‐synthesized PBFDO (before and after dialysis) (Figure 3a–d) reveal that PBFDO chains are primarily oriented edge‐on to the substrate. Notably, TMQ‐synthesized PBFDO (before dialysis) exhibits a more convoluted diffraction pattern, with several peaks in both the out‐of‐plane and in‐plane directions, which suggests the formation of highly crystalline aggregates of TMQ and TMQH.^[^ ^64^, ^65^ ^]^ In contrast, no α‐TQ aggregates are visible in the diffraction pattern of α‐TQ‐synthesized PBFDO (before dialysis). A strong π–π stacking (010) peak located at *q*_xy_ = 1.85 Å^−1^ (*d*‐spacing = 3.39 Å) is observed, with a peak position comparable to that of dialyzed PBFDO samples synthesized using either α‐TQ or TMQ (Figure 3f). α‐TQ‐synthesized PBFDO (before dialysis) displays a slightly shorter π–π stacking coherence length (20.3 Å) compared to dialyzed PBFDO synthesized using TMQ (23.4 Å) and a slightly larger paracrystalline disorder (0.154 vs 0.143, see Figures S6–S8, Supporting Information). A larger lamellar (100) packing distance of 11.57 Å (*q*_z_ = 0.543 Å^−1^) is observed in α‐TQ‐synthesized PBFDO before dialysis, compared to 10.80 Å (*q*_z_ = 0.582 Å^−1^) in dialyzed TMQ‐synthesized PBFDO (Figure 3e and Figure S6, Supporting Information). This difference is likely due to residual α‐TQ/α‐TOH in the films, which induces a slight expansion of the PBFDO unit cell.^[^ ^66^ ^]^ Atomic force microscopy (AFM), carried out on thin films of PBFDO synthesized using either TMQ or α‐TQ (before and after dialysis), reveals the presence of large TMQ aggregates in the TMQ‐synthesized PBFDO (before dialysis) films (Figure 3g). In stark contrast, α‐TQ‐synthesized PBFDO films display a smooth surface morphology, regardless of the dialysis steps, similar to that of dialyzed PBFDO polymerized with TMQ (Figure 3h–j).

### 2.3 Electrical and Mechanical Characterization

**Figure 4:**
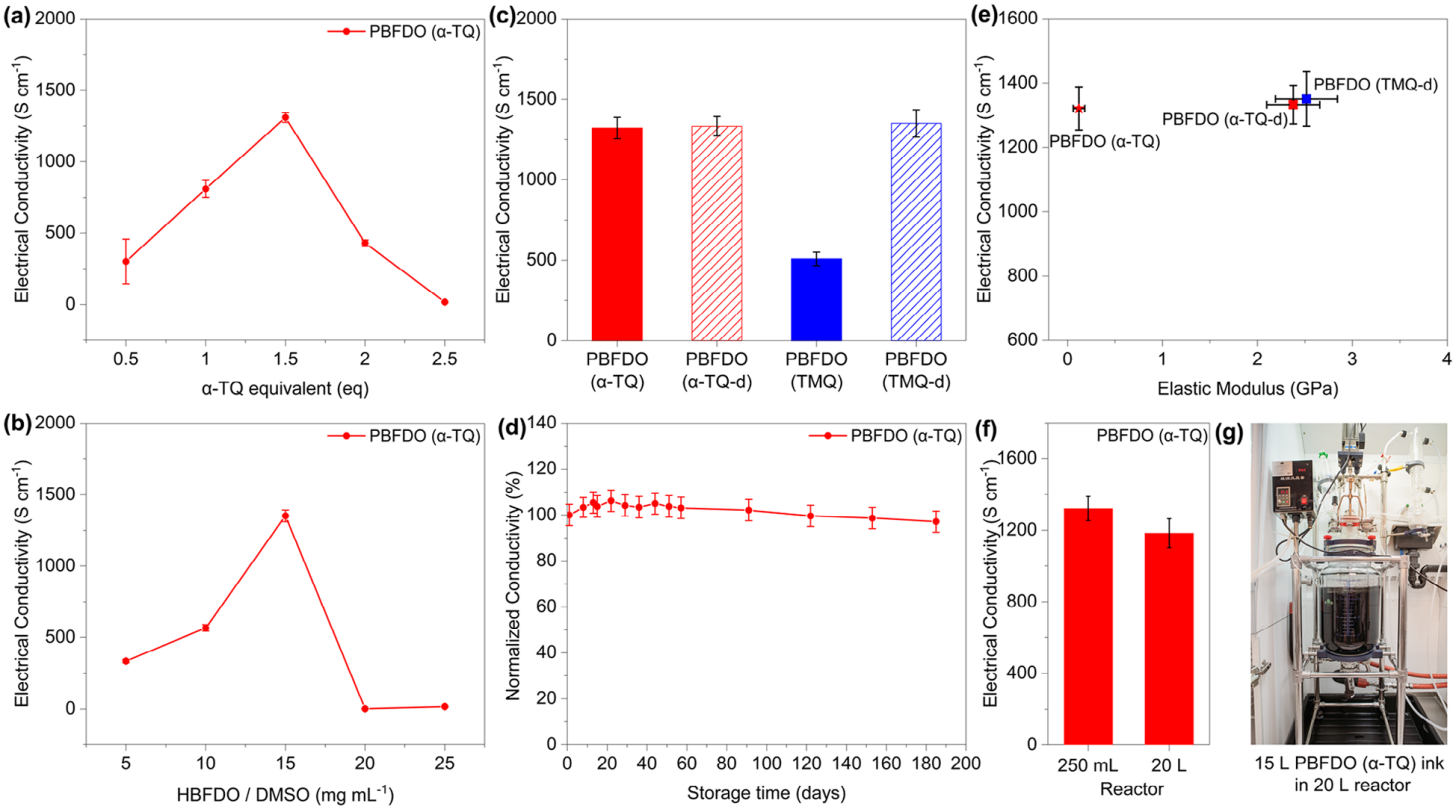
a) Electrical conductivity of α‐TQ‐synthesized PBFDO (before dialysis) as a function of α‐TQ catalyst loading. b) Electrical conductivity of α‐TQ‐synthesized PBFDO (before dialysis) as a function of HBFDO monomer concentration. c) Electrical conductivity of α‐TQ‐synthesized PBFDO (before and after dialysis) and TMQ‐synthesized PBFDO (before and after dialysis). The conductivity values were determined by measuring at least three films for each sample. d) Normalized electrical conductivity of α‐TQ‐synthesized PBFDO (before dialysis) thin films over time under ambient conditions. e) Comparison of electrical conductivity and elastic modulus for α‐TQ‐synthesized PBFDO (before and after dialysis) and TMQ‐synthesized PBFDO (after dialysis). f) Electrical conductivity of PBFDO (before dialysis) synthesized in both 250 mL and 20 L reactors. For the 250 mL reactor, three batches of PBFDO (α‐TQ) were synthesized, with three devices measured per batch. For the 20 L reactor, one batch was synthesized, and six devices were tested. g) Photograph of a 15 L α‐TQ‐synthesized PBFDO ink inside the 20 L reactor.

We evaluated the effect of catalyst and monomer concentration on the electrical properties of PBFDO polymerized using α‐TQ (**Figure** **4**). We found that the electrical conductivity varies significantly with the amount of α‐TQ catalyst (Figure 4a) and HBFDO monomer (Figure 4b), reaching about 1300 S cm^−1^ at 1.5 eq α‐TQ and 15 mg mL^−1^ HBFDO. The low electrical conductivity observed at low HBFDO/α‐TQ concentrations is attributed to a lower degree of polymerization, consistent with previous observations for TMQ‐synthesized PBFDO.^[^ ^52^ ^]^ In contrast, the drop in electrical conductivity at high monomer/catalyst concentrations is ascribed to gelation during polymerization, leading to inhomogeneous films after spin coating (Figure S9, Supporting Information). Solutions of PBFDO synthesized with 1.5 eq α‐TQ and 15 mg mL^−1^ HBFDO exhibit good wettability on various substrates, including glass, silicon, and polyethylene terephthalate (PET), ensuring uniform film formation (Figure S10, Supporting Information).

Remarkably, α‐TQ‐synthesized PBFDO exhibits electrical conductivity values that are independent of the dialysis process, with values reaching 1321 ± 67 S cm^−1^ before dialysis and 1333 ± 60 S cm^−1^ after dialysis, respectively (Figure 4c). These values are comparable to those measured for TMQ‐synthesized PBFDO after dialysis (1351 ± 85 S cm^−1^) and in line with previous reports based on the TMQ catalyst.^[^ ^32^, ^48^ ^]^ In stark contrast, the electrical conductivity of TMQ‐synthesized PBFDO is highly dependent on the dialysis process, with values around 508 ± 42 S cm^−1^ before dialysis. This demonstrates that α‐TQ offers a significant advantage over TMQ by eliminating the need for lengthy purification steps while still preserving high electrical conductivity.

To demonstrate the potential technological implications for large‐scale synthesis, we carried out a 15 L batch polymerization of PBFDO with α‐TQ in a 20‐L reactor (Figure 4g and Figure S11, Supporting Information). HBFDO (225 g, 1.18 mol) and α‐TQ (792.87 g, 1.77 mol) were dissolved in 15 L of DMSO, and the reactor was heated to 100 °C for 1.5 h, then cooled to room temperature after polymerization. The PBFDO solution was collected from the bottom of the reactor and used directly for conductivity measurements without further purification, showing a conductivity of approximately 1183 ± 81 S cm^−1^, on par with that of PBFDO synthesized in the 250 mL reactor (Figure 4f). The α‐TQ‐synthesized PBFDO films show excellent ambient stability, retaining over 97% of their initial electrical conductivity even after being exposed to air for 180 days (Figure 4d). This remarkable stability aligns with the high work function values determined by ultraviolet photoelectron spectroscopy measurements, which were found to be >4.82 eV (Figure S12, Supporting Information), in agreement with previous reports.^[^ ^48^, ^52^ ^]^ Despite the significant presence of residual α‐TQ/α‐TOH in the films, α‐TQ‐synthesized PBFDO also demonstrates exceptional thermal and photostability (Figure S13, Supporting Information).

We then investigated the elastic modulus of PBFDO synthesized using α‐TQ (before and after dialysis) and via the TMQ route. The elastic modulus of the films was estimated by indenting at a minimum of 20 positions for each film, including dialyzed TMQ‐synthesized PBFDO and α‐TQ‐synthesized PBFDO before and after dialysis (Figure S14, Supporting Information). The average elastic modulus values for α‐TQ‐synthesized PBFDO before dialysis, after dialysis, and TMQ‐synthesized PBFDO after dialysis were 0.12 GPa, 2.4 GPa, and 2.5 GPa, respectively (Figure 4e). The order‐of‐magnitude difference in the elastic modulus of α‐TQ‐synthesized PBFDO before and after dialysis is likely due to residual α‐TQ/α‐TOH in the PBFDO film, which acts as a plasticizer by weakening intermolecular interactions between PBFDO chains. This is consistent with the observed expansion of the PBFDO unit cell upon α‐TQ/α‐TOH inclusion (Figure 3e) and aligns with studies on α‐TQ‐blended polyethylene, where α‐TQ has been reported to increase polymer chain mobility.^[^ ^67^ ^]^ A similar drop in elastic modulus has been observed for PEDOT:PSS in the presence of plasticizers (Figure S15, Supporting Information). The removal of α‐TQ/α‐TOH during dialysis enhances molecular ordering, as evidenced by increased coherence lengths in both lamellar and π‐π stacking distances (Figure S8b, Supporting Information), resulting in a more rigid PBFDO film. Additionally, α‐TQ‐synthesized PBFDO exhibits good thermal stability with no observable phase transition (Figures S16, S17, Supporting Information). Note that we refer to α‐TQ/α‐TOH as a plasticizer because it reduces stiffness and enhances processability, similar to plasticizing additives used in the polymer industry. However, due to its relatively high content (≈79 wt%), the material should be considered a liquid/polymer composite rather than a conventional plasticized polymer.

### 2.4 Thermoelectric Performance

**Figure 5:**
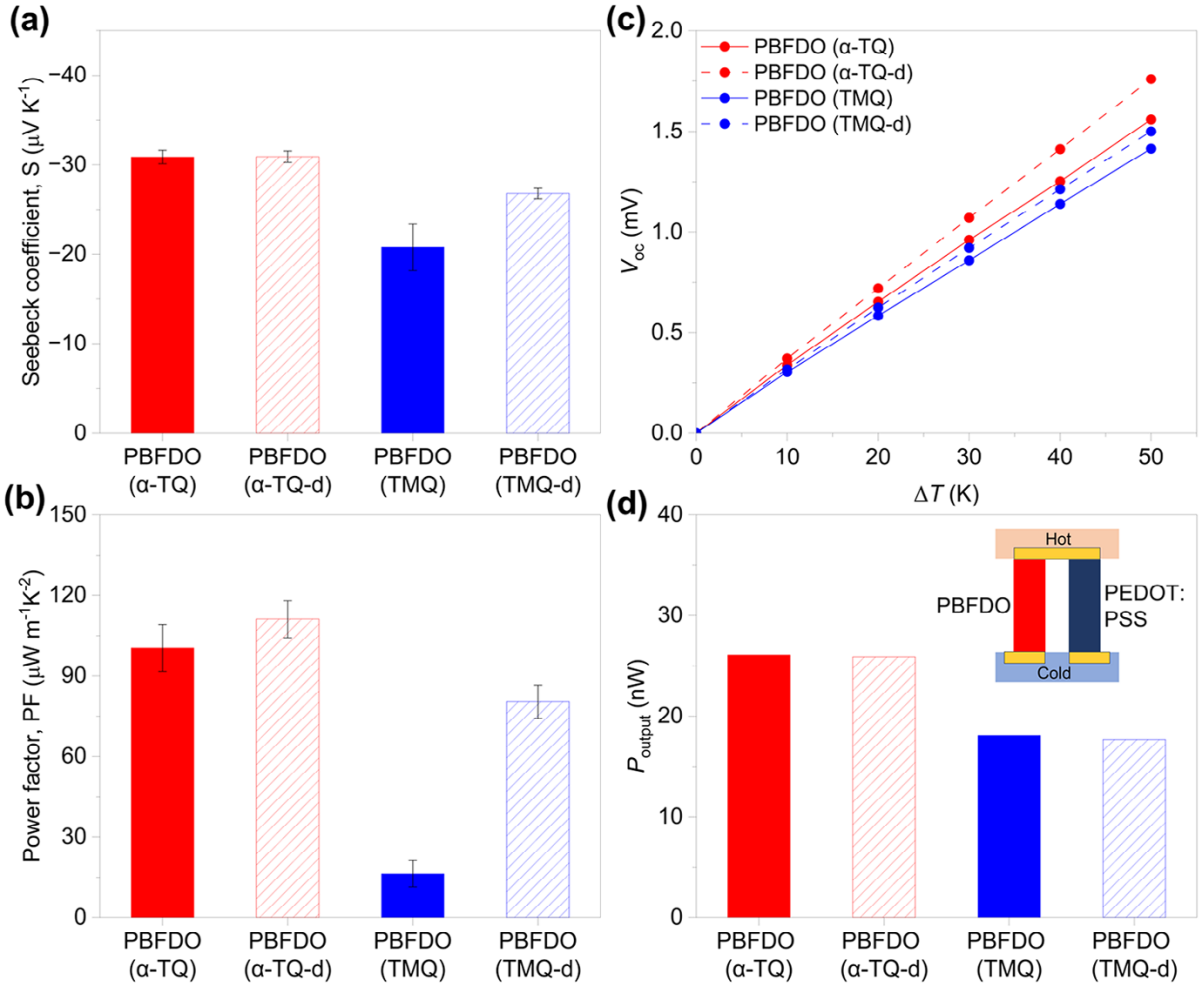
a) Seebeck coefficient (*S*), b) power factor (*PF*), and c) open‐circuit voltage (*V*_OC_) of α‐TQ‐synthesized PBFDO (before and after dialysis) and TMQ‐synthesized PBFDO (before and after dialysis). d) Output power of thermocouples composed of one p‐leg PEDOT:PSS and one n‐leg α‐TQ‐synthesized PBFDO (before and after dialysis) or TMQ‐synthesized PBFDO (before and after dialysis) at the same temperature gradient (Δ*T* = 50 K).

Next, we evaluated the thermoelectric properties of PBFDO synthesized using α‐TQ and compared them to the conventional TMQ‐synthesized PBFDO. The Seebeck coefficient (*S*) of α‐TQ‐synthesized PBFDO shows values around ‐30.9 ± 0.8 µV K^−1^ before dialysis and ‐30.9 ± 0.7 µV K^−1^ after dialysis. These values are slightly larger than those measured for TMQ‐synthesized PBFDO, which were measured to be ‐20.8 ± 2.6 µV K^−1^ before dialysis and ‐26.8 ± 0.6 µV K^−1^ after dialysis. The negative sign of the Seebeck coefficient confirms the n‐type character of PBFDO (**Figure** **5**a and Figure S18, Supporting Information). The resulting power factor (*PF* = *σS* ^2^) reaches values of 100.4 ± 8.8 µW m^−1^ K^−2^ for α‐TQ‐synthesized PBFDO before dialysis and 111.2 ± 6.9 µW m^−1^ K^−2^ for α‐TQ‐synthesized PBFDO after dialysis (Figure 5b and Table S1, Supporting Information), which are larger than those measured for TMQ‐synthesized PBFDO (16.4 ± 4.9 µW m^−1^ K^−2^ before dialysis and 80.4 ± 6.2 µW m^−1^ K^−2^ after dialysis, Figure 5b). These values are among the highest reported for n‐type thermoelectric polymers (Table S2, Supporting Information). In addition, the presence of residual α‐TQ/α‐TOH significantly reduces the in‐plane thermal conductivity by more than one order of magnitude. Using the beam‐offset frequency‐domain thermoreflectance method,^[^ ^68^ ^]^ we measured a decrease in thermal conductivity (*κ*) for TMQ‐synthesized PBFDO from 6.04 W m^−1^ K^−1^ after dialysis (4.04 W m^−1^ K^−1^ before dialysis) to 0.48 W m^−1^ K^−1^ for α‐TQ‐synthesized PBFDO before dialysis (6.84 W m^−1^ K^−1^ after dialysis) (Figure S19a, Supporting Information). The resulting dimensionless figure of merit, *zT*  = σ*S* ^2^ *T*/κ , increases from 0.004 for TMQ‐synthesized PBFDO after dialysis to a maximum of approximately 0.068 for α‐TQ‐synthesized PBFDO before dialysis (Figure S19b, Supporting Information). These results demonstrate that the incorporation of α‐TQ enables the development of a thermoelectric composite with significantly reduced thermal conductivity, making it a promising approach for optimizing rigid conducting polymers.

With these high *PF* values at hand, we fabricated flexible all‐polymer organic thermocouples using α‐TQ‐synthesized PBFDO (without dialysis) as the n‐leg and PEDOT:PSS [5 vol % ethylene glycol (EG), *S* = 22.3 ± 0.6 µV K^−1^, *σ* = 1081 ± 46 S cm^−1^] as the p‐leg. These flexible thermocouples exhibit open‐circuit voltages and short‐circuit currents that are linearly proportional to the temperature gradient, with an internal resistance of approximately 23 Ω (Figure 5c and Figures S20a, S21a, Supporting Information). The power output per p‐n pair of the thermocouple, which follows a square relationship with the temperature gradient (Δ*T*), ranges from 1.26 nW (Δ*T* = 10 K) to 26 nW (Δ*T* = 50 K) (Figure S22a, Supporting Information). These values are larger than those measured for equivalent thermocouples comprising dialyzed PBFDO polymerized using TMQ (Figure 5d and Figure S22, Supporting Information).

## 3 Conclusions

In summary, we have demonstrated that using α‐TQ eliminates the need for post‐reaction dialysis in PBFDO synthesis. The unique structure of α‐TQ prevents crystallization upon reduction to α‐TOH, allowing the catalyst to remain in solution without compromising film formation. This yields PBFDO thin films with high electrical conductivity (1321 ± 67 S cm^−1^), comparable to that of dialyzed TMQ‐synthesized PBFDO (1351 ± 85 S cm^−1^), while also exhibiting excellent thermal, photo, and air stability, retaining 97% of their conductivity after 180 d in ambient conditions. The Seebeck coefficient of α‐TQ‐synthesized PBFDO remained stable at approximately ‐30.9 µV K^−1^ before and after dialysis, yielding a power factor exceeding 100 µW m^−1^ K^−2^ and outperforming TMQ‐synthesized PBFDO. When integrated into flexible thermocouples with PEDOT:PSS as the p‐type leg, power outputs reached up to 26 nW at Δ*T* = 50 K. Additionally, residual α‐TQ/α‐TOH acts as a plasticizer, reducing the elastic modulus of α‐TQ‐synthesized PBFDO by more than an order of magnitude compared to TMQ‐synthesized films while preserving high electrical conductivity. This unique combination of mechanical and electrical properties is particularly notable, as additives typically lower the elastic modulus of conducting polymers at the expense of electrical conductivity (Figure S14, Supporting Information). This makes PBFDO suitable for mechanically compliant organic electronics and electronic textiles.^[^ ^69^ ^]^ Furthermore, α‐TQ‐synthesized PBFDO exhibits significantly lower thermal conductivity than dialyzed TMQ‐synthesized PBFDO while maintaining a high Seebeck coefficient, making it a promising thermoelectric material with high conversion efficiency. Finally, the successful upscaling in a 20 L reactor demonstrates the technological relevance of this approach, enabling the production of high‐conductivity n‐type PBFDO ink that is ready for use without dialysis.

## 4 Experimental Section

### Materials

Tetramethyl‐1,4‐benzoquinone (TMQ) was purchased from Tokyo Chemical Industry (TCI). 1,4‐Benzoquinone (BQ), ethyl cyanoacetate, absolute ethanol, ammonia solution 28%, hydrochloric acid 37%, activated charcoal, toluene, acetic anhydride (Ac_2_O), tetrahydrofuran (THF), chloroform, rac‐α‐tocopherol (α‐TOH), diethyl ether, iron (III) chloride hexahydrate, and methanol were purchased from Sigma‐Aldrich and were used without further purification.

### Synthesis of HBFDO

3,7‐dihydrobenzo[1,2‐b:4,5‐b′]difuran‐2,6‐dione (HBFDO) was synthesized following a reported procedure.^[^ ^70^ ^]^ In brief, two dropping funnels were affixed to a three‐necked flask (Flask A), which was immersed in a circulating water bath to maintain a constant temperature. Ethyl cyanoacetate (21.26 g, 20 mL, 187.94 mmol), ethanol (66 mL), and concentrated ammonium hydroxide (15.01 g, 16.66 mL, 431.35 mmol) were then added to Flask A. Meanwhile, benzoquinone (36 g, 333.04 mmol) and ethanol (266 mL) were introduced into a separate round‐bottomed flask (Flask B) and stirred at 40 °C for 30 min. Following this, ethyl cyanoacetate (37.51 g, 35.29 mL, 331.61 mmol) was added to Flask B and transferred to one of the dropping funnels while hot. Concentrated ammonium hydroxide (60.32 g, 66.6 mL, 1724.37 mmol) was diluted with water (100 mL) and transferred to the second dropping funnel. The reagents from both dropping funnels were added simultaneously, with the drop rate carefully controlled. When approximately 10% of the benzoquinone solution remained, the ammonia solution was completely added. The entire addition process lasted approximately 30 min. Upon formation of a purple‐red precipitate, the reaction mixture was stirred for an additional hour. The resulting solid was collected via suction filtration, washed thoroughly with ethanol, and dried to yield 22.13 g of diethyl 2,2′‐(2,5‐dihydroxy‐1,4‐phenylene)bis(2‐cyanoacetate) (DHPDCA) as a dark purple solid (20% yield).

For the subsequent hydrolysis step, DHPDCA (25 g, 75 mmol), concentrated hydrochloric acid (172.5 g, 145 mL, 1750 mmol), and water (130 mL) were added to a round‐bottomed flask and refluxed at 110 °C overnight. After completion, water (125 mL) and activated charcoal (5 g) were added to the hot reaction mixture, which was then boiled for another 5 min. The mixture was filtered through a funnel to remove the activated charcoal, and the residue was washed with a small volume of hot water. The resulting filtrate was cooled, leading to the formation of white crystalline 2,2′‐(2,5‐dihydroxy‐1,4‐phenylene)diacetic acid (DHPDA), which was obtained in a yield of 12.25 g (72%).

In the final step, DHPDA (16 g, 70.74 mmol) was dissolved in anhydrous toluene (800 mL), followed by the addition of acetic anhydride (172.8 g, 160 mL, 1690 mmol). The reaction mixture was stirred at 100 °C overnight, after which the solvent was removed under vacuum. The resulting residue was treated with methanol and filtered. The obtained solid was then dissolved in acetonitrile by stirring at 90 °C. The solution was subsequently cooled in an ice bath to induce crystallization. The resulting product, HBFDO, was collected as white crystals (12.11 g, 90% yield; Figure S1a in the Supporting Information).

### Synthesis of α‐Tocopherylquinone (α‐TQ)

α‐TQ was synthesized following a reported procedure.^[^ ^62^ ^]^ α‐TOH (10 g) was dissolved in diethyl ether (100 mL) and mixed with ferric chloride hexahydrate solution (2 g in 25 mL of methanol/water, 50/50 V/V). The mixture was stirred for 30 min, after which the organic phase was separated. This process was repeated six times with fresh portions of ferric chloride hexahydrate solution. The organic phase was then washed with water three times and dried over sodium sulfate. The solvent was removed using a rotary evaporator, yielding α‐TQ as a yellow oil (9.4 g, 91%; Figure 1b).

### Synthesis of PBFDO

PBFDO synthesized using the TMQ catalyst followed a previously reported procedure.^[^ ^48^ ^]^ For the synthesis of PBFDO using α‐TQ, HBFDO (3.18 g, 16.72 mmol) and α‐TQ (11.21 g, 25.08 mmol) were dissolved in DMSO (212 mL) under a nitrogen atmosphere. The mixture was then heated to 100 °C and stirred for 1 h, yielding an α‐TQ‐synthesized PBFDO solution with a solid content of approximately 13 mg mL^−1^. The yield of PBFDO polymerization catalyzed by α‐TQ was in the range of 80–85%, as measured over six batches. Dialysis of both α‐TQ‐synthesized and TMQ‐synthesized PBFDO was performed following previous reports.^[^ ^48^ ^]^ The elemental analysis of TMQ‐synthesized PBFDO and α‐TQ‐synthesized PBFDO after dialysis is reported in Table S5 of the Supporting Information.

The intrinsic viscosity ([*η*]) of α‐TQ‐synthesized PBFDO (after dialysis) and TMQ‐synthesized PBFDO (after dialysis) was measured using an Ubbelohde viscometer, yielding values of 9.287 dL g^−1^ and 8.182 dL g^−1^, respectively. Since [*η*] is related to the molecular weight (*M*) of a polymer through the Mark‐Houwink equation ([*η*] = *K M^a^*), where *K* and *a* are specific to the polymer‐solvent system and temperature, and these conditions were the same for both samples, α‐TQ‐synthesized PBFDO and TMQ‐synthesized PBFDO have similar molecular weights.

### Thin‐Film Casting

Thin films of PBFDO (synthesized via α‐TQ or TMQ) were fabricated by spin‐coating (1500 rpm, 120 s, acceleration 1500 rpm s^−1^, then 3000 rpm, 10 s, acceleration 3000 rpm s^−1^) the solution onto prewashed and plasma‐cleaned substrates like glass, Si or Si/SiO_2._ The films were dried on top of a hot plate at 40 °C.

### UV‐Vis‐Near Infrared and FTIR

α‐TQ‐synthesized PBFDO (before and after dialysis) and TMQ‐synthesized PBFDO (after dialysis) were deposited on glass substrates by spin‐coating. Their UV–Vis–near infrared spectra were measured by Perkin Elmer Lambda 900. All the FTIR samples except α‐TQ and α‐TOH were prepared by evaporating solvent to form solid samples on a 70 °C hotplate and measured using PerkinElmer Spectron 3 FT‐IR spectrometer in ATR mode.

### X‐Ray Photoelectron Spectroscopy (XPS)

The samples were spin‐coated onto a silicon substrate with evaporated Cr/Au (5/50 nm) and then transferred into the load lock chamber of the ultrahigh vacuum system for the following tests. XPS measurements were performed with a Scienta ESCA 200 hemispherical analyzer using a monochromatized Al Kα source with a photon energy of 1486.6 eV. The measurements were carried out with a base pressure lower than 1 × 10^−9^ mbar.

### Ultraviolet Photoelectron Spectroscopy (UPS)

UPS experiments were conducted using a Scienta ESCA 200 system under a base pressure of 2 × 10^−10^ mbar, equipped with an SES 200 electron analyzer and a helium discharge lamp (*hv* = 21.22 eV). All spectra were recorded at normal emission and room temperature. Binding energies were referenced to the Fermi level (0.0 eV). The work function was determined from the secondary electron cutoff after applying a ‐3 V bias to the sample.

### Grazing‐Incidence Wide‐Angle X‐Ray Scattering and AFM Characterization

All samples were deposited on silicon wafers for GIWAXS measurements. The samples were measured at Beamline 9A in the Pohang Accelerator Laboratory in South Korea. The X‐ray energy was 11.08 eV and the incidence angle was 0.12°. The samples were measured in vacuum, and the total exposure time was 10 s. The scattered X‐rays were recorded by a charge‐coupled device detector located 220.8498 mm from the sample. AFM images were recorded with an Icon XR from Bruker, using a silicon nitride cantilever with a spring constant of 40 N m^−1^.

### Electrical Characterization

Electrical conductivity was measured by a four‐point probe method using a Keithley 4200‐SCS semiconductor characterization system. The conductivity was calculated using the formula $\sigma =\frac{IL}{VWd}\;$, where *I* is the current, *V* is the voltage, *W* is the sample width, *d* is the film thickness, and *L* is the length between two electrodes. In this study, *W* = 7 mm, *L* = 3 mm, *d* depends on different samples.

### Mechanical Properties Characterization

Three 1 in. × 0.5 in. glass substrates were sonicated in an acetone bath and an IPA bath for 8 min each. 1000 µL of α‐TQ‐synthesized PBFDO (before dialysis), α‐TQ‐synthesized PBFDO (after dialysis), and TMQ‐synthesized PBFDO (after dialysis) inks were drop‐casted in two runs (500 µL in each run) on the respective glass substrate at 40 °C. After solvent evaporation, the films were placed in a vacuum oven at 40 °C for up to 48 h to remove solvent traces, if any. Nanoindentation was carried out by indenting at room temperature and relative humidity of about 31% (α‐TQ‐synthesized PBFDO (before dialysis) and dialyzed TMQ‐synthesized PBFDO films) and 42% (dialyzed α‐TQ‐synthesized PBFDO films) with a Hysitron TI Premier instrument from Bruker. The nanoindenter is equipped with a Berkovich tip made of diamond with a half angle of 65.27°, calibrated with a reference quartz substrate. Prior to each experiment, the instrument was left in idle condition for one hour to reach thermal equilibrium. The maximum drift for all experiments was set to 0.02 nm s^−1^, resulting in an error in indentation depth of less than 0.5%. The elastic modulus was extracted from the holding segment of the load–displacement curve using creep analysis.^[^ ^71^ ^]^ The elastic modulus was evaluated at a minimum of 20 locations on each film.

### Organic Thermocouples

The thermocouples had a p/n‐leg pair module designed with an in‐plane geometry on the 100 µm thick polyethylene naphthalate (PEN) substrate. PEDOT:PSS (PH1000) treated with ethylene glycol (EG 5 vol%) was used for the p‐leg, while PBFDO was used for the n‐leg. The thickness values of both p‐ and n‐legs are provided in Table S3 in the Supporting Information. The length and width of the p/n‐leg were set to 2.5 mm and 2 mm, respectively. First, Cr/Au (5 nm/ 50 nm) were deposited onto the PEN substrate by evaporation through a shadow mask. Then, PBFDO and PEDOT:PSS were drop‐cast on the PEN substrate in air. The samples were then dried on a hot plate at 40 °C. The contributions to the internal resistance were determined as 20% (PEDOT:PSS p‐leg), 30% (PBFDO n‐leg), 12% (electrodes), and 38% (contact resistance) (Table S4, Supporting Information). All fabrication processes and measurements were conducted in air without encapsulation.

### Thermal Conductivity

Ring‐shaped polydimethylsiloxane (PDMS) molds (height = 1.2 mm, inner diameter = 3.5 mm) were placed in conformal contact with the substrates (low‐stress SiN*_x_*, Norcada), centered on the transparent 0.5 × 0.5 mm^2^ membrane (thickness = 10 nm). Aliquots of PBFDO inks were pipetted into the molds, with the concentration and volume (4‐8 µL) determining the resulting film thickness. Samples were then dried at 40 °C under blanket N_2_ flow for 1.5–3 h. The PDMS mold pinned the edges of drop‐cast inks, preventing their contraction and yielding homogeneous film areas over the membrane after solvent evaporation. Film thicknesses were determined by absorption spectroscopy using calibration data for absorbance of the ≈488 nm peak versus thickness measured on reference films on glass substrates. Then, the samples were coated with a 5 nm thick gold layer on the silicon nitride (SiN_*x*_) side. The effective thermal conductance (*G*_total_, where *G*_total_ = *G*_film_ + *G*_substrate_) of the sample was derived using the measured thickness and the specific heat capacity and density values of each layer. The substrate thermal conductance (*G*_substrate_) was obtained from a blank test substrate, i.e., a 10 nm SiN*_x_* layer with a 5 nm gold coating. By subtracting *G*_substrate_ from *G*_total_, the thermal conductance of the organic film *G*_film_ was calculated. Finally, the in‐plane thermal conductivity of the film (*κ*_film_) was determined based on the measured film thickness.

### General Materials Characterization

Nuclear magnetic resonance (NMR) measurements were performed on a 500 MHz Bruker NMR workstation. Thermogravimetric analysis (TGA) was performed on a TA Q500, and the samples were heated from 50 °C to 850 °C with a heating rate of 10 °C min^−1^ under a nitrogen atmosphere. Differential scanning calorimetry (DSC) was tested by TA DSC250 under nitrogen flow at heating/cooling rates of 10/10 °C min^−1^. The samples of α‐TQ‐synthesized PBFDO after dialysis and TMQ‐synthesized PBFDO after dialysis used for TGA and DSC measurements were dialyzed and washed with THF and chloroform in a Soxhlet extractor overnight, respectively.

## Conflict of Interest

J.‐D.H., Q.L., M.‐A.S., C.‐Y.Y., and S.F. have filed a provisional patent application related to this work (no. PCT/EP2024/063710). M.‐A.S., C.‐Y.Y., and S.F. are the co‐founders of n‐Ink AB. Q.W., M.‐A.S., and N.P. are employees of n‐Ink AB. The other authors declare that they have no conflict of interest.

## Supporting information



Supporting Information

## Data Availability

The data that support the findings of this study are available from the corresponding author upon reasonable request.
