# Supplementary material for: Preventing Benzoquinone‐Based Catalyst Aggregation Enables the One‐Step Synthesis of Highly Conductive Poly(benzodifurandione) without Post‐Reaction Purification
Source: Adv Mater. 2025 Mar 18;37(17):2502426. doi: 10.1002/adma.202502426 (PMC12038537; doi:10.1002/adma.202502426)
Supplement: Supplementary file 1 — Supporting Information [file ADMA-37-2502426-s001.pdf]

ADVANCED MATERIALS

Supporting Information

for *Adv. Mater.*, DOI 10.1002/adma.202502426

Preventing Benzoquinone-Based Catalyst Aggregation Enables the One-Step Synthesis of Highly Conductive Poly(benzodifurandione) without Post-Reaction Purification

Jun-Da Huang, Qifan Li, Qingqing Wang, Tiefeng Liu, Sang Young Jeong, Sri Harish Kumar Paleti, Tom P. A. van der Pol, Kai Xu, Han-Yan Wu, Natalie Pinchin, Marc-Antoine Stoeckel, Wenlong Jin, Aleksandr Perevedentsev, Xianjie Liu, Juan Sebastián Reparaz, Mariano Campoy-Quiles, Han Young Woo, Christian Müller, Mats Fahlman, Chi-Yuan Yang and Simone Fabiano**

Supporting Information

Preventing Benzoquinone-Based Catalyst Aggregation Enables the One-Step Synthesis of Highly Conductive Poly(Benzodifurandione) Without Post-Reaction Purification

Jun-Da Huang,[‡] *Qifan Li,*[‡] *Qingqing Wang, Tiefeng Liu, Sang Young Jeong, Sri Harish Kumar Paleti, Tom P. A. van der Pol, Kai Xu, Han-Yan Wu, Natalie Pinchin, Marc-Antoine Stoeckel, Wenlong Jin, Aleksandr Perevedentsev, Xianjie Liu, Juan Sebastián Reparaz, Mariano Campoy-Quiles, Han Young Woo, Christian Müller, Mats Fahlman, Chi-Yuan Yang* and Simone Fabiano**

J.-D. Huang, Q. Li, T. Liu, T. P. A. van der Pol, H.-Y. Wu, M.-A. Stoeckel, W. Jin, X. Liu, M. Fahlman, C.-Y. Yang, S. Fabiano
Laboratory of Organic Electronics, Department of Science and Technology, Linköping University, SE-60174 Norrköping, Sweden.
E-mail: chi-yuan.yang@liu.se; simone.fabiano@liu.se

J.-D. Huang, M. Fahlman, S. Fabiano
Wallenberg Wood Science Center, Department of Science and Technology, Linköping University, SE-60174 Norrköping, Sweden.

Q. Wang, N. Pinchin, M.-A. Stoeckel, C.-Y. Yang, S. Fabiano
n-Ink AB, Källvindsgatan 5, SE-60240 Norrköping, Sweden

T. Liu, M.-A. Stoeckel, S. Fabiano
Wallenberg Initiative Materials Science for Sustainability, Department of Science and Technology, Linköping University, SE-60174 Norrköping, Sweden.

S. Y. Jeong, H. Y. Woo
Department of Chemistry, College of Science, Korea University, Seoul 136-713, Republic of Korea.

S. H. K. Paleti, C. Müller
Department of Chemistry and Chemical Engineering, Chalmers University of Technology, 41296 Göteborg, Sweden

K. Xu, A. Perevedentsev, J. S. Reparaz, M. Campoy-Quiles,
Department of Nanostructured Materials, Institut de Ciència de Materials de Barcelona, ICMAB-CSIC, E-08193 Bellaterra, Spain

C. Müller
Wallenberg Wood Science Center, Chalmers University of Technology, 41296 Göteborg, Sweden

C. Müller
Stellenbosch Institute for Advanced Study, Wallenberg Research Centre at Stellenbosch University, 7600 Stellenbosch, South Africa

[‡] J.-D.H. and Q.L. contributed equally to this work.

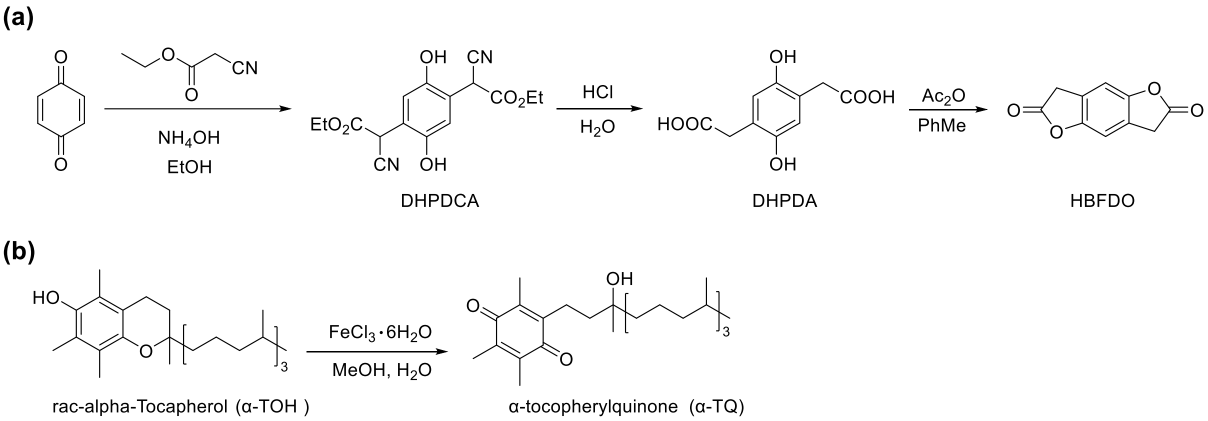

Figure S1. a) Synthesis of HBFDO. b) Synthetic of α-TQ.

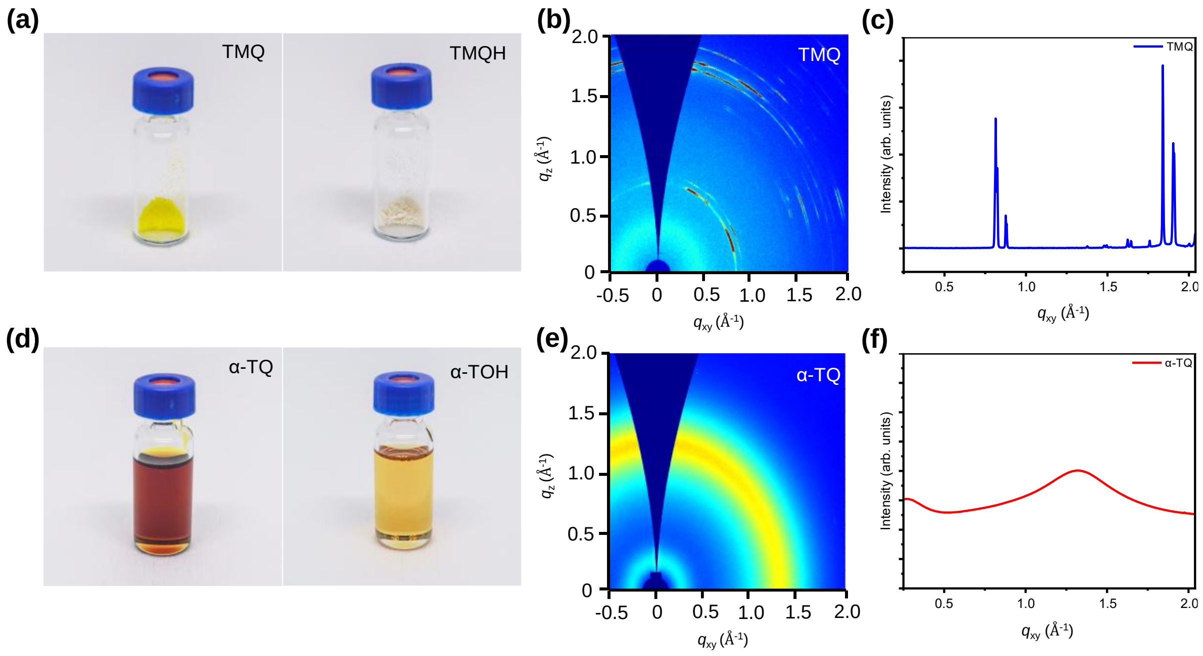

Figure S2. a) Photographs of TMQ and TMQH. b) 2D WAXS pattern and c) 1D linecuts of TMQ. d) Photographs of α-TQ and α-TOH. e) 2D WAXS pattern and f) 1D linecuts of α-TQ.

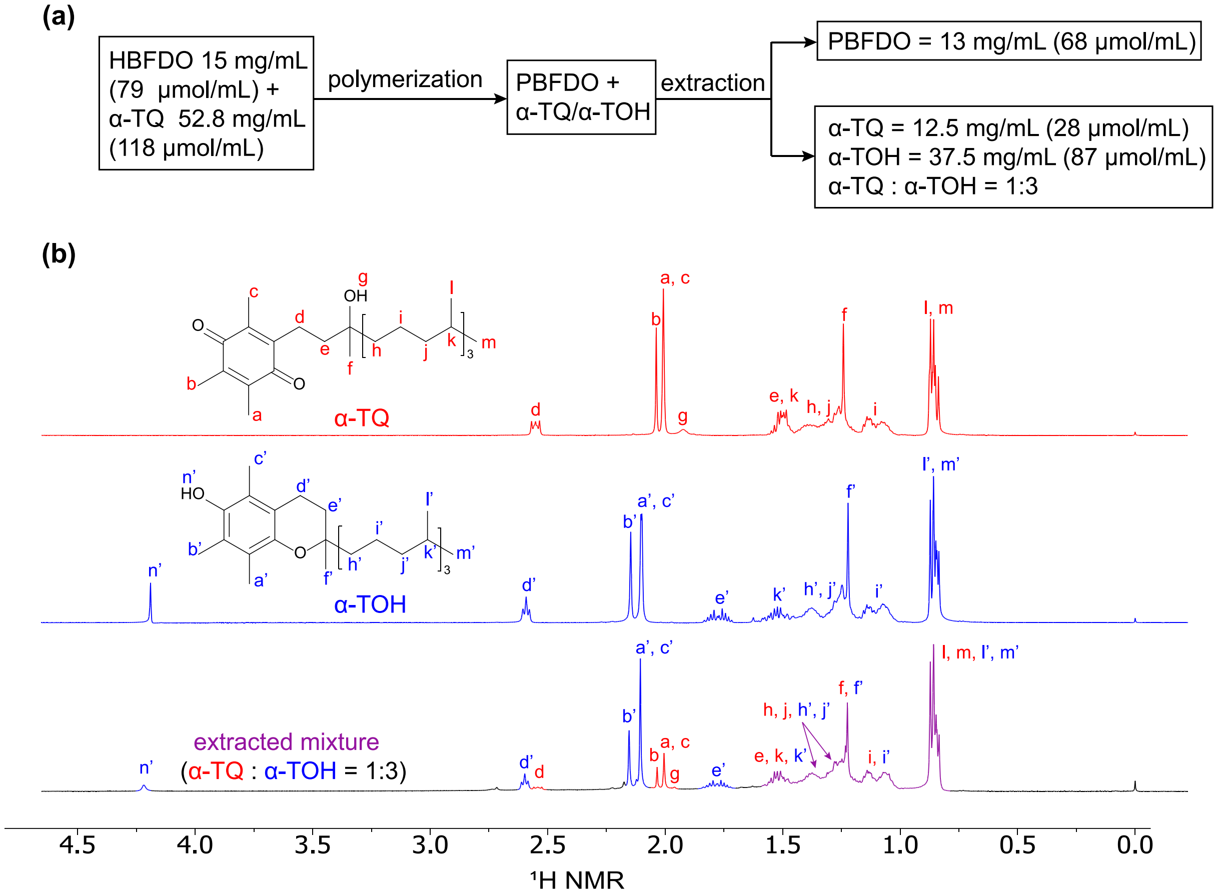

Figure S3. a) Flowchart of the catalyst extraction process. b) ^{1}H NMR of α-TQ, α-TOH, and the extracted mixture. Based on NMR integration, the ratio of α-TQ to α-TOH in the extracted mixture was determined to be 1:3.

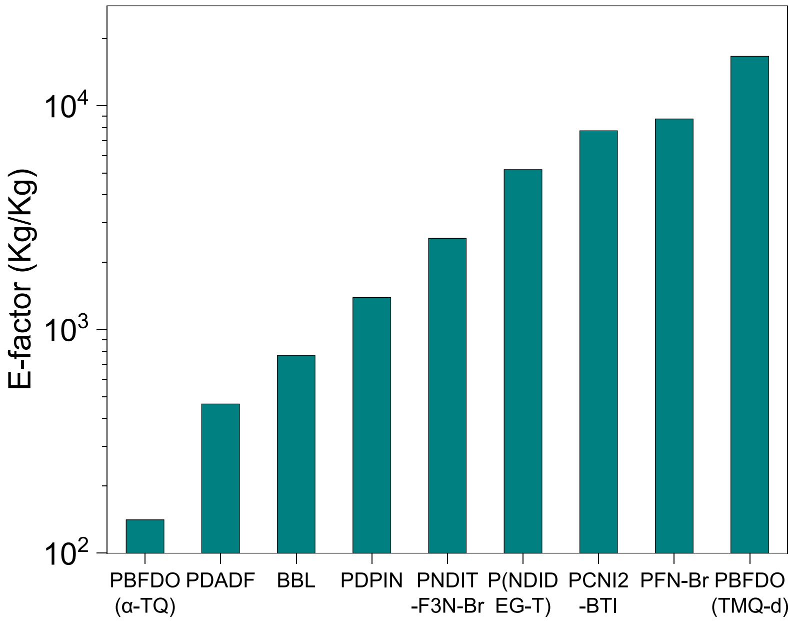

Figure S4. E-factor for the synthesis of α-TQ-synthesized PBFDO (before dialysis) and comparison with TMQ-synthesized PBFDO (after dialysis) and other known n-type polymers (note: only polymerization and purification steps were considered here).[1–8]

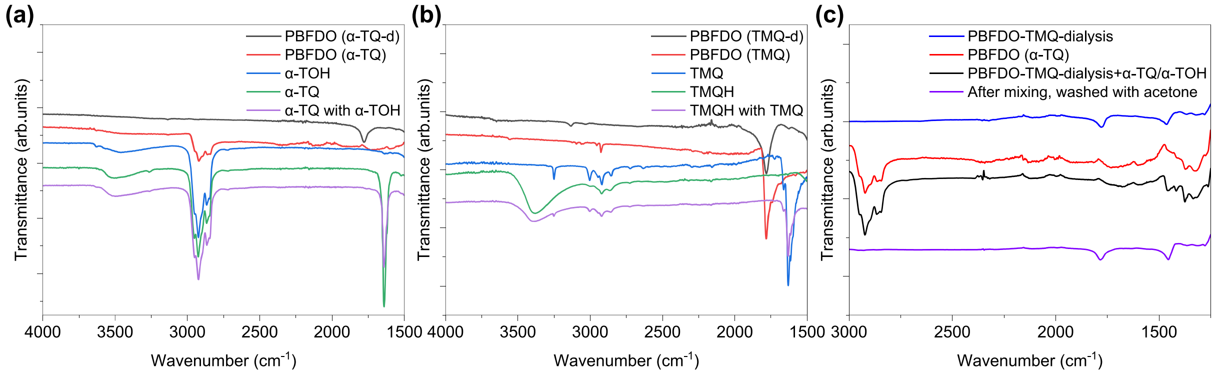

Figure S5. a) FTIR spectra of α-TQ-synthesized PBFDO with and without α-TQ, confirming the absence of α-TQ/α-TOH after dialysis. b) FTIR spectra of TMQ-synthesized PBFDO with and without TMQ, demonstrating the absence of TMQ/TMQH after dialysis. c) FTIR spectra comparing dialyzed TMQ-synthesized PBFDO, α-TQ-synthesized PBFDO (without dialysis), dialyzed TMQ-synthesized PBFDO mixed with 1.5 eq of α-TQ/α-TOH (1:3 mass ration), and the latter after washing with acetone. The FTIR spectrum of pure dialyzed TMQ-synthesized PBFDO (blue line) exhibits characteristic peaks at 1781 cm^{-1} and 1450 cm^{-1}. After mixing with α-TQ and α-TOH, the resulting spectrum (black line) closely matches that of α-TQ-synthesized PBFDO (red line), where the fine structure of the PBFDO signal below 2000 cm^{-1} was masked, while the characteristic peaks of α-TQ and α-TOH (2750-3000 cm^{-1}) remained visible. Upon washing the mixture film with acetone, the characteristic PBFDO peaks reappeared (purple line), aligning with those of dialyzed TMQ-synthesized PBFDO.

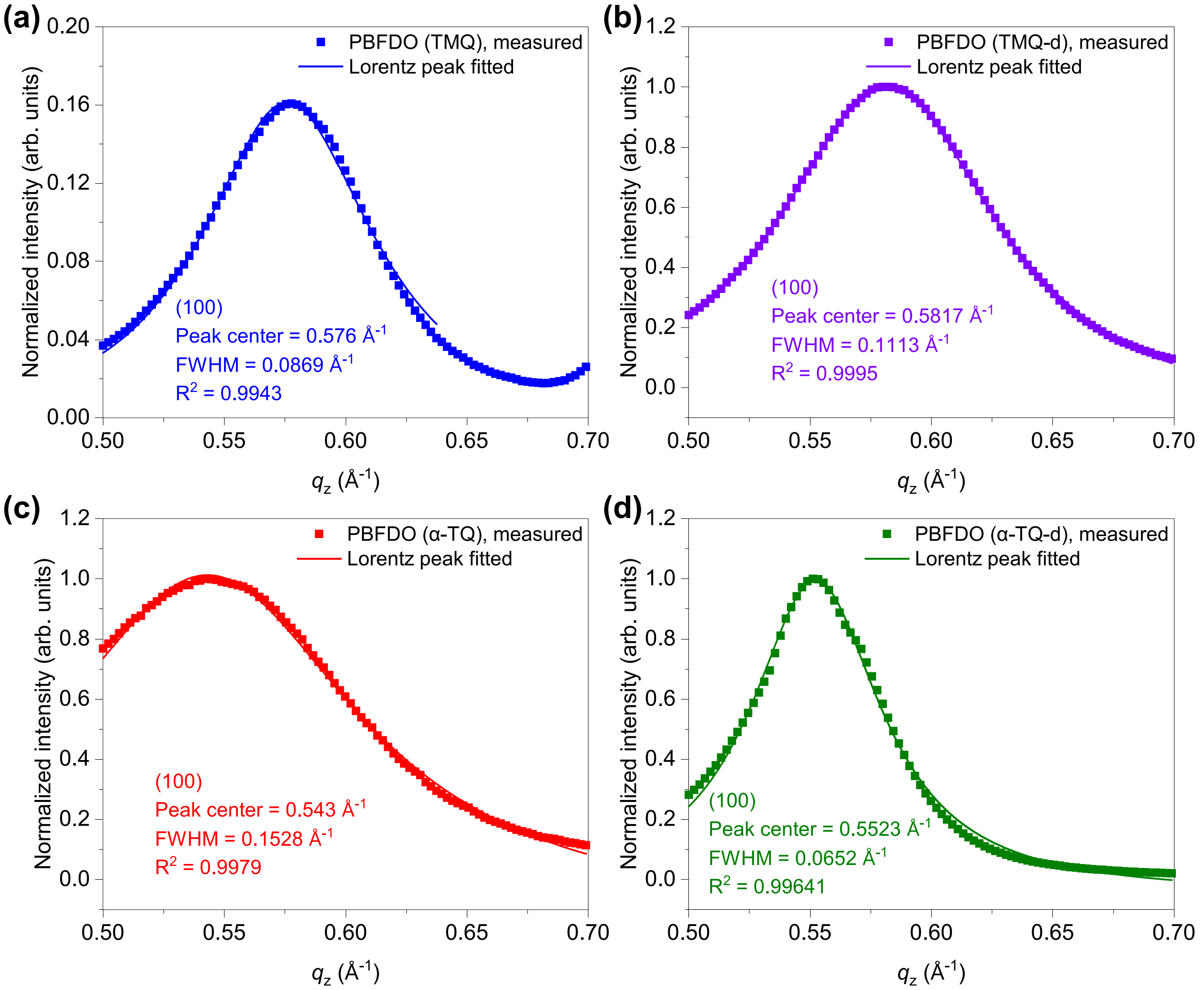

Figure S6. Lamellar (100) diffraction analysis of a) TMQ-synthesized PBFDO (before dialysis), b) TMQ-synthesized PBFDO (after dialysis), c) α-TQ-synthesized PBFDO (before dialysis), and d) α-TQ-synthesized PBFDO (after dialysis).

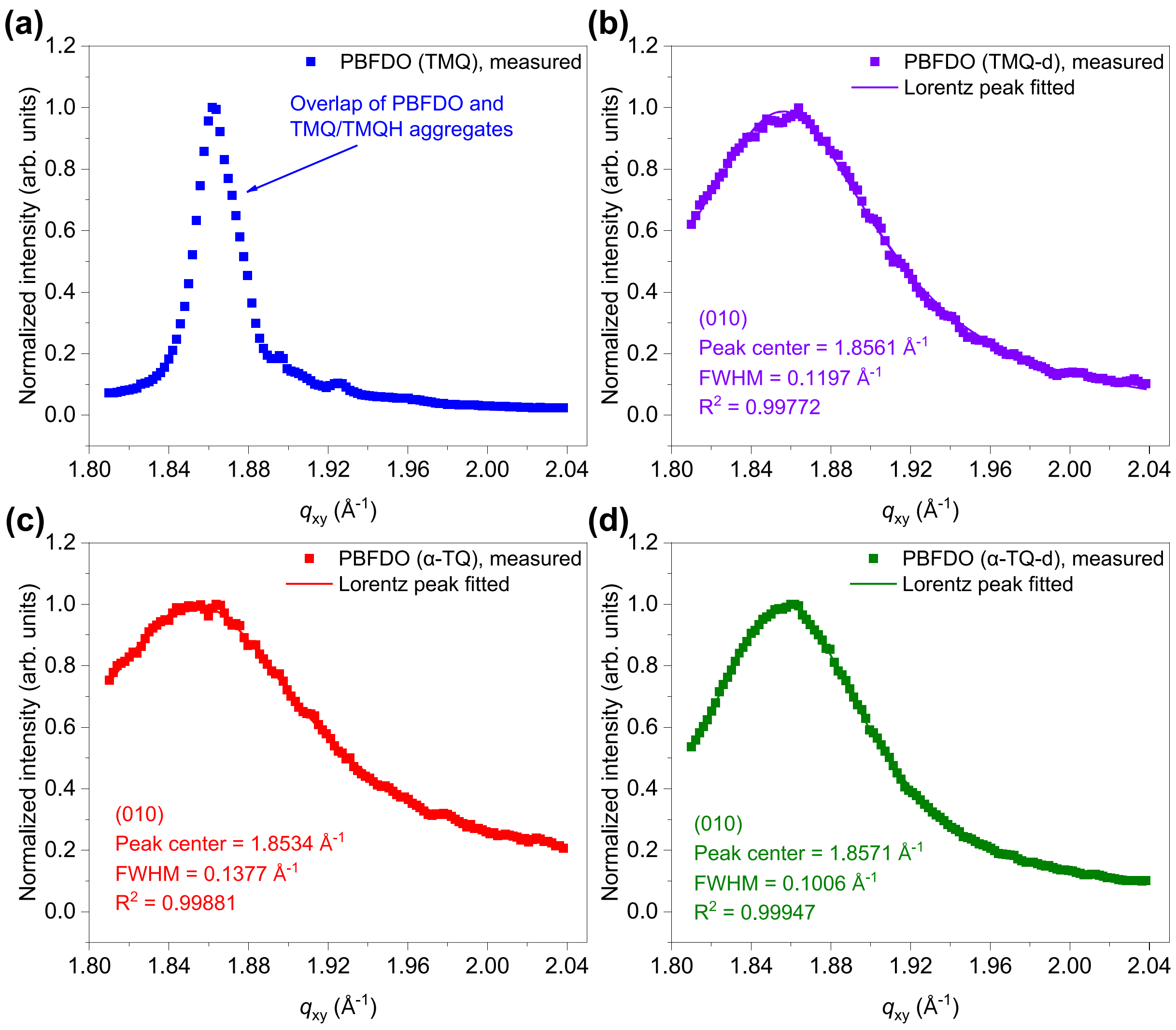

Figure S7. π-π stacking (010) diffraction analysis of a) TMQ-synthesized PBFDO (before dialysis), b) TMQ-synthesized PBFDO (after dialysis), c) α-TQ-synthesized PBFDO (before dialysis), d) and α-TQ-synthesized PBFDO (after dialysis).

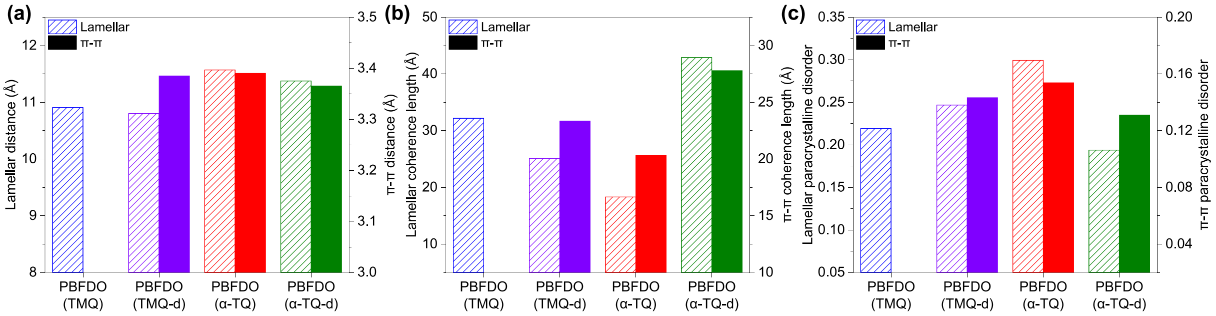

Figure S8. a) Summary of the calculated lamellar and π-π stacking distances, b) lamellar and π-π stacking coherence lengths, and c) lamellar and π-π stacking paracrystalline disorder as extracted from GIWAXS 1D data of TMQ-synthesized PBFDO (before and after dialysis) and α-TQ-synthesized PBFDO (before and after dialysis).

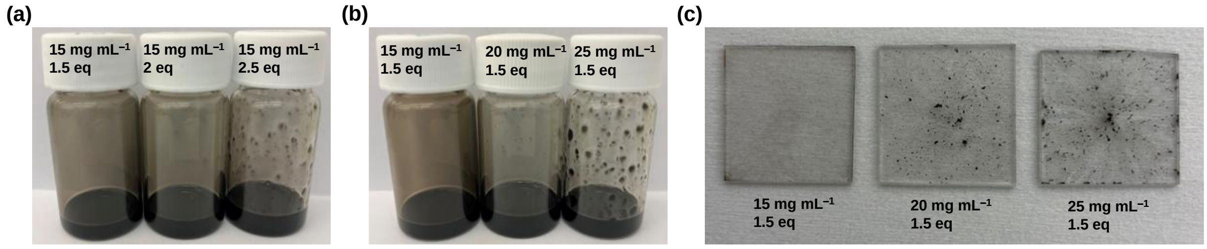

Figure S9. a) α-TQ-synthesized PBFDO (before dialysis) prepared with a constant HBFDO monomer concentration (15 mg mL^{-1}) and varying α-TQ catalyst equivalents (1.5 eq, 2 eq, and 2.5 eq). b) α-TQ-synthesized PBFDO (before dialysis) prepared with a constant α-TQ catalyst equivalent (1.5 eq) and varying HBFDO monomer concentrations (15 mg mL^{-1}, 20 mg mL^{-1}, and 25 mg mL^{-1}). c) Thin films of α-TQ-synthesized PBFDO (before dialysis) spin-coated on glass substrates under different synthesis conditions.

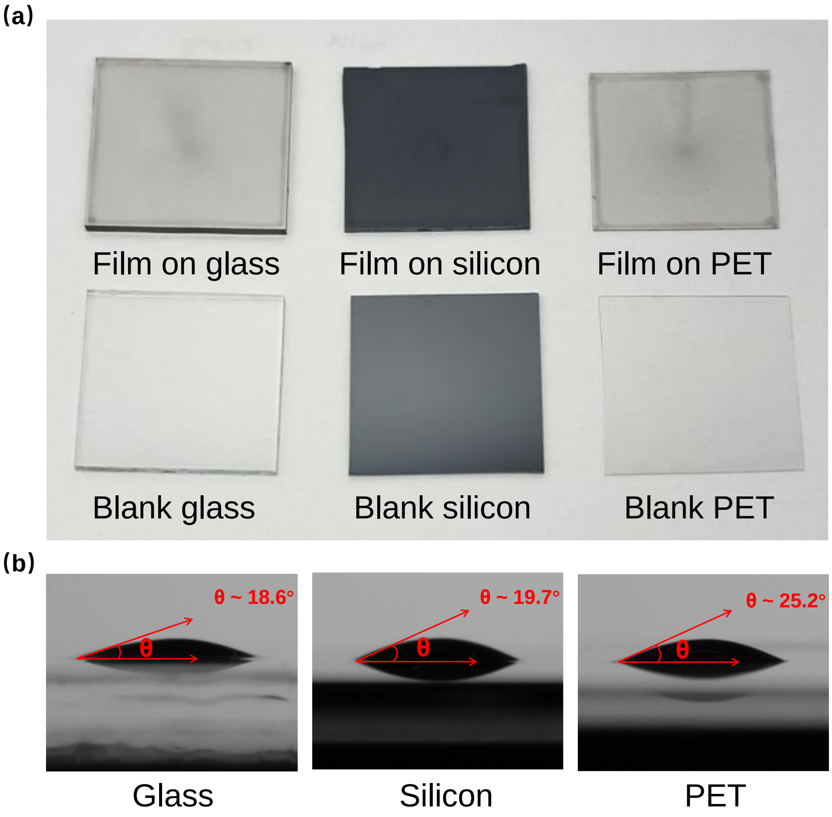

Figure S10. a) α-TQ-synthesized PBFDO films deposited on glass, silicon, and polyethylene terephthalate (PET) substrates (2.5 × 2.5 cm). b) Contact angle measurements of the α-TQ-synthesized PBFDO solution on these substrates.

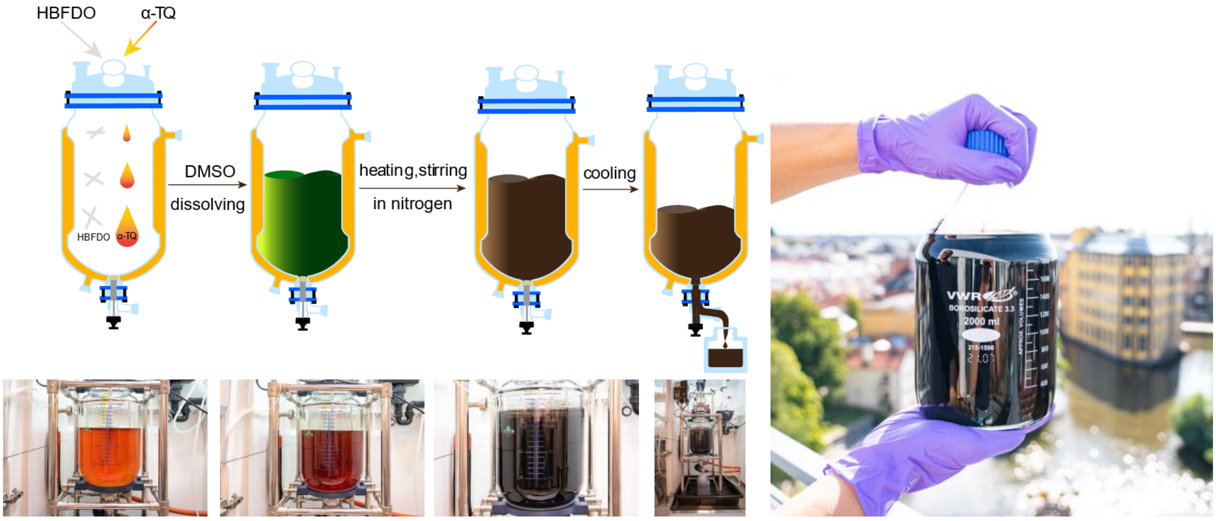

Figure S11. Schematic diagram and photographs of the 15-L α-TQ-synthesized PBFDO ink.

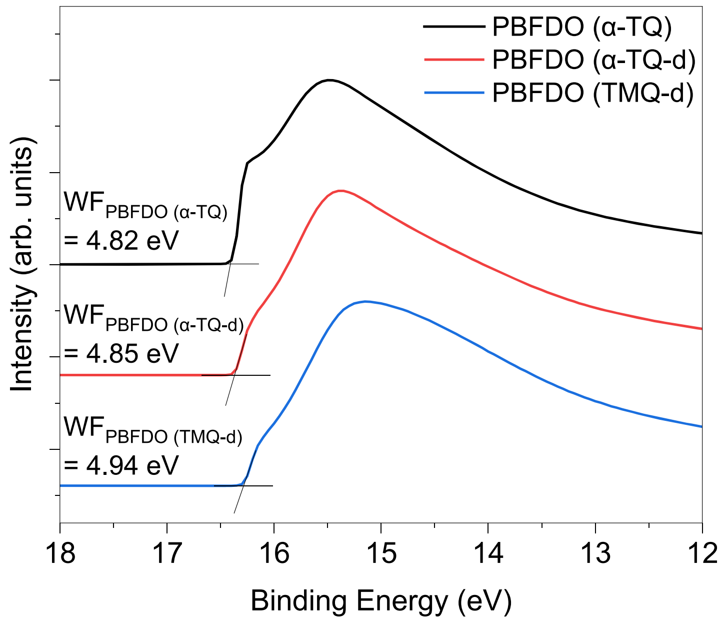

Figure S12. UPS analysis of PBFDO synthesized with different catalysts.

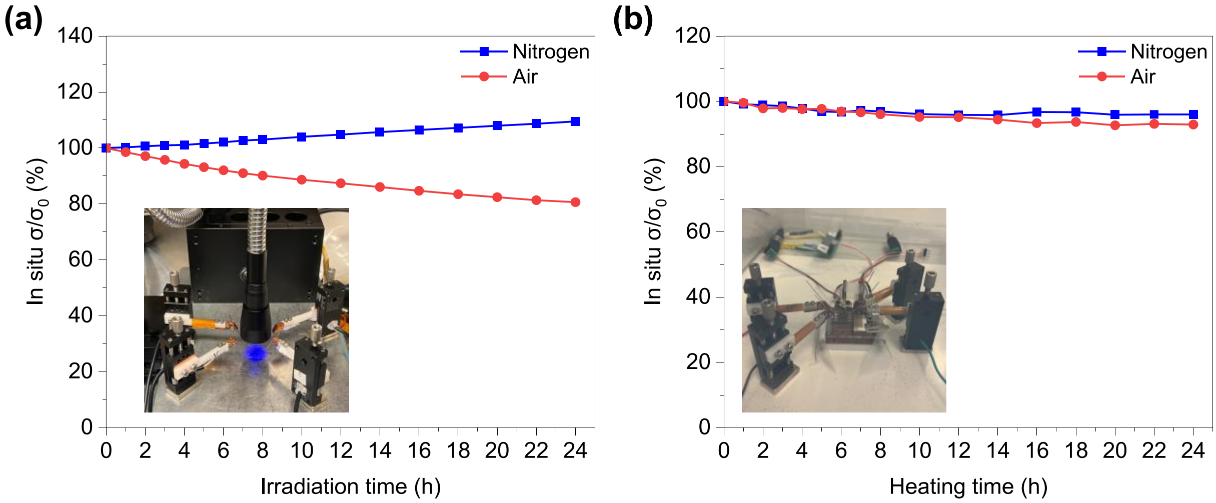

Figure S13. a) Photostability of α-TQ-synthesized PBFDO, measured by irradiating the films with 395 nm UV light at 100 mW cm^{-2} for 24 hours in both air and nitrogen atmosphere. In nitrogen, the conductivity increased by ~10%, while in air, it decreased by ~20%. b) Thermal stability of α-TQ-synthesized PBFDO, measured by heating the films at 100 °C for 24 hours in air and nitrogen. The conductivity remained at 96% of the initial values in nitrogen and 93% in air, demonstrating excellent thermal stability.

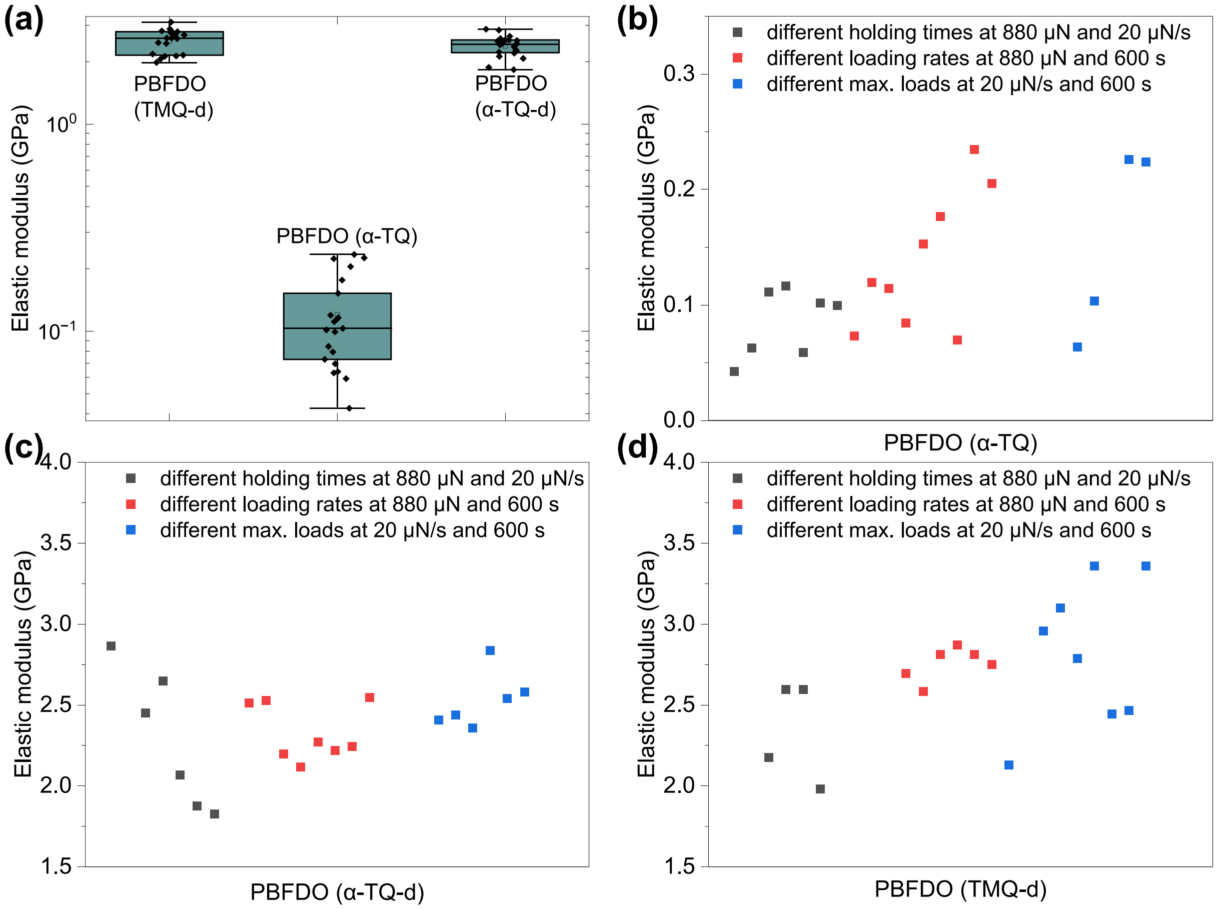

Figure S14. a) Box-whisker plot summarizing the elastic modulus of α-TQ-synthesized PBFDO (before dialysis), α-TQ-synthesized PBFDO (after dialysis), and TMQ-synthesized PBFDO (after dialysis) films. Elastic modulus measurements of b) α-TQ-synthesized PBFDO (before dialysis), c) α-TQ-synthesized PBFDO (after dialysis), and d) TMQ-synthesized PBFDO (after dialysis).

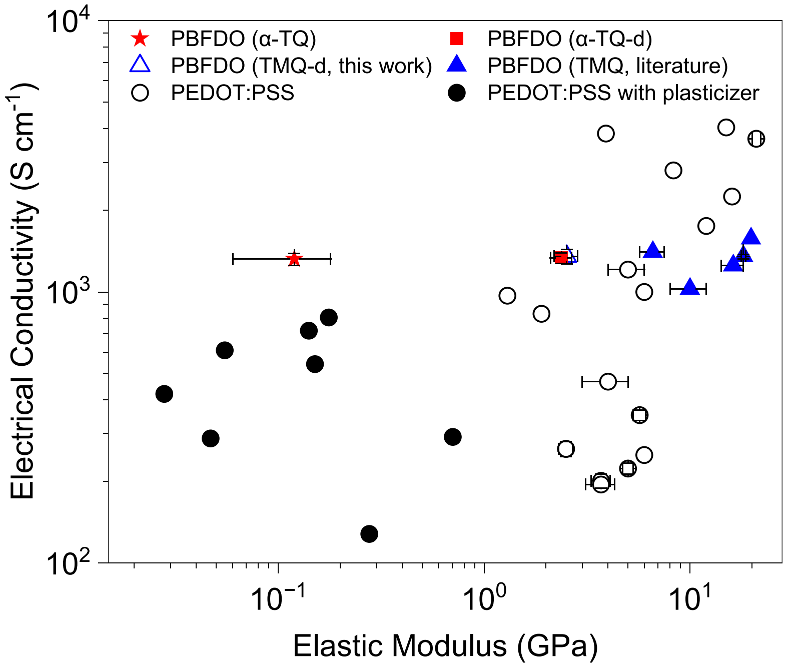

Figure S15. Comparison of electrical conductivity and elastic modulus of α-TQ-synthesized PBFDO (before and after dialysis) with TMQ-synthesized PBFDO (both from this work and as reported in the literature[9,10]) and PEDOT:PSS (with and without plasticizers, as reported in the literature[11–24]). The electrical conductivity of α-TQ-synthesized PBFDO is comparable to that of TMQ-synthesized PBFDO despite having an elastic modulus >10× lower. Compared to PEDOT:PSS with similar electrical conductivity, α-TQ-synthesized PBFDO exhibits an elastic modulus at least 10× lower. While plasticizers can reduce the elastic modulus of PEDOT:PSS to values similar to α-TQ-synthesized PBFDO, this typically results in lower conductivity.

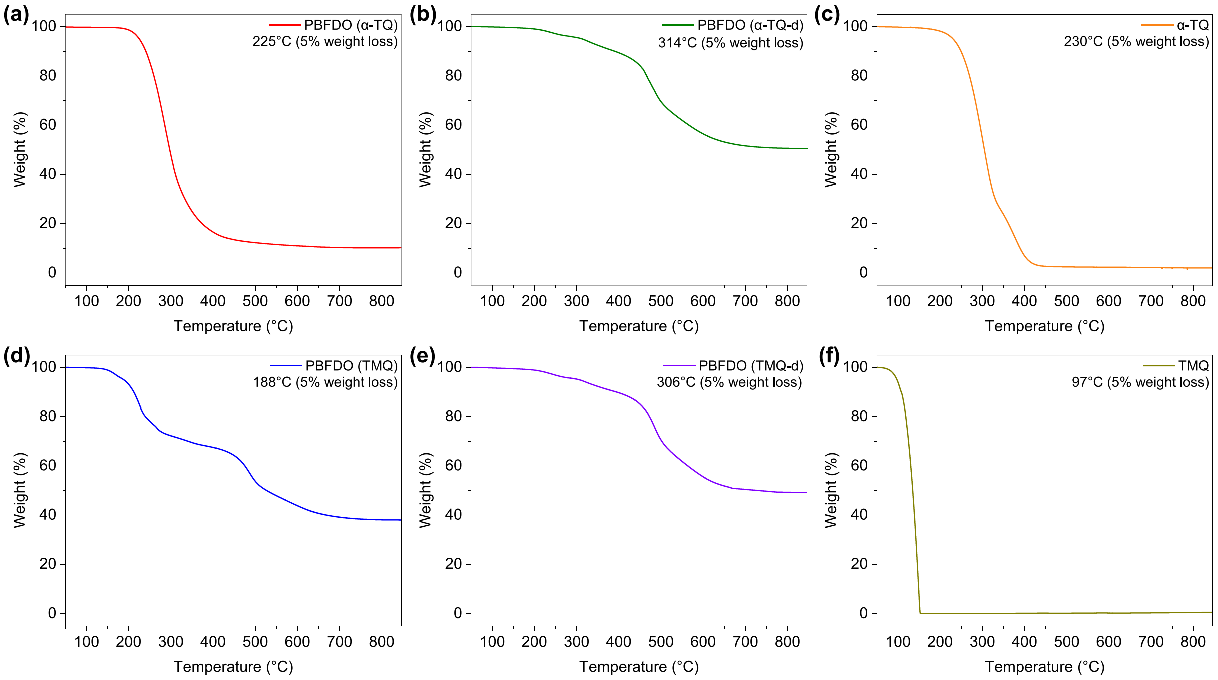

Figure S16. Thermogravimetric analysis of a) α-TQ-synthesized PBFDO (before dialysis), b) α-TQ-synthesized PBFDO (after dialysis), c) α-TQ, d) TMQ-synthesized PBFDO (before dialysis), e) TMQ-synthesized PBFDO (after dialysis) and f) TMQ.

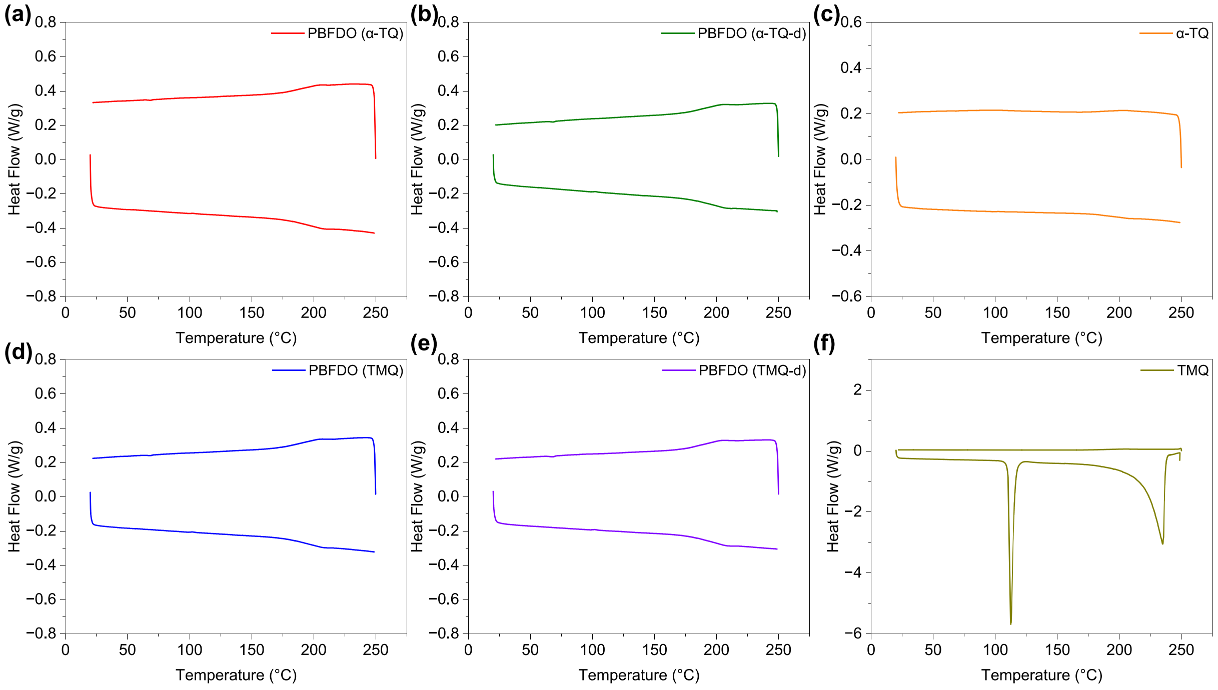

Figure S17. Differential scanning calorimetry (DSC) curves of a) α-TQ-synthesized PBFDO (before dialysis), b) α-TQ-synthesized PBFDO (after dialysis), c) α-TQ, d) TMQ-synthesized PBFDO (before dialysis), e) TMQ-synthesized PBFDO (after dialysis) and f) TMQ. All curves exhibited no obvious exothermic or endothermic behavior in the range of 25 ~ 250 °C except for TMQ.

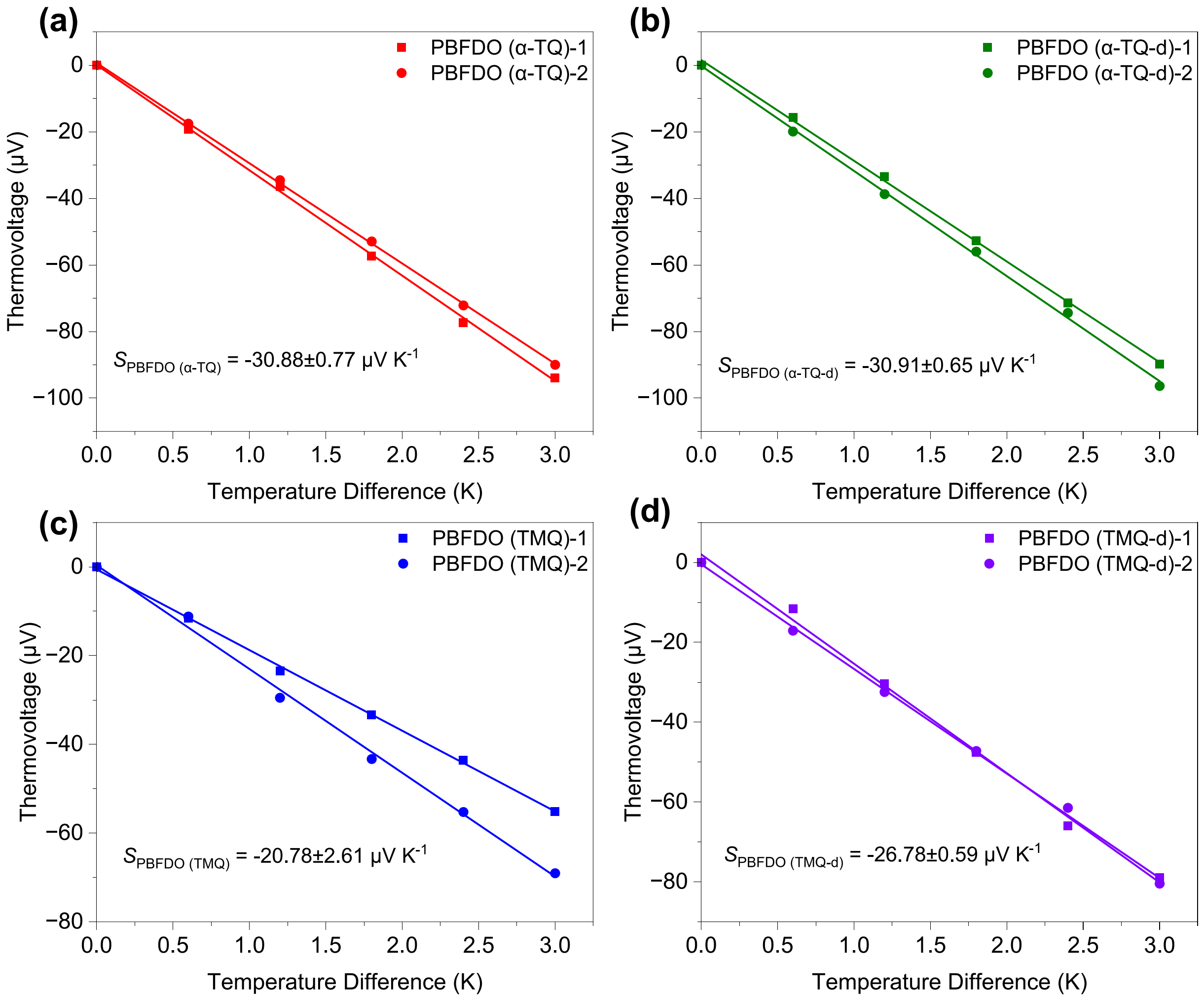

Figure S18. Seebeck coefficient (*S*) of a) α-TQ-synthesized PBFDO (before dialysis), b) α-TQ-synthesized PBFDO (after dialysis), c) TMQ-synthesized PBFDO (before dialysis), and d) TMQ-synthesized PBFDO (after dialysis). The negative value indicates the n-type character of these polymers.

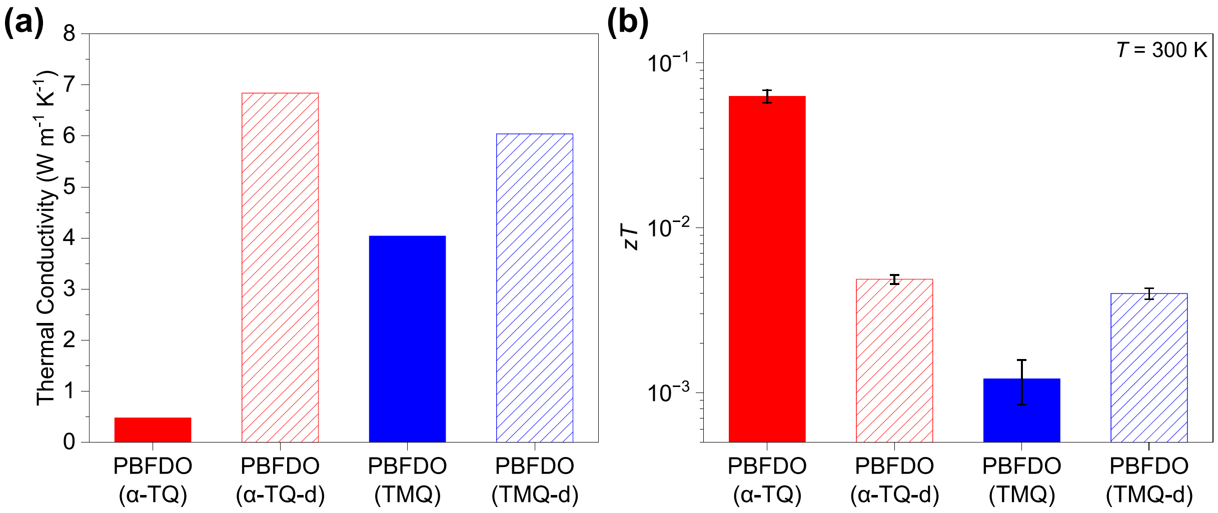

Figure S19. a) Thermal conductivity and b) *zT* of PBFDO synthesized using various catalysts.

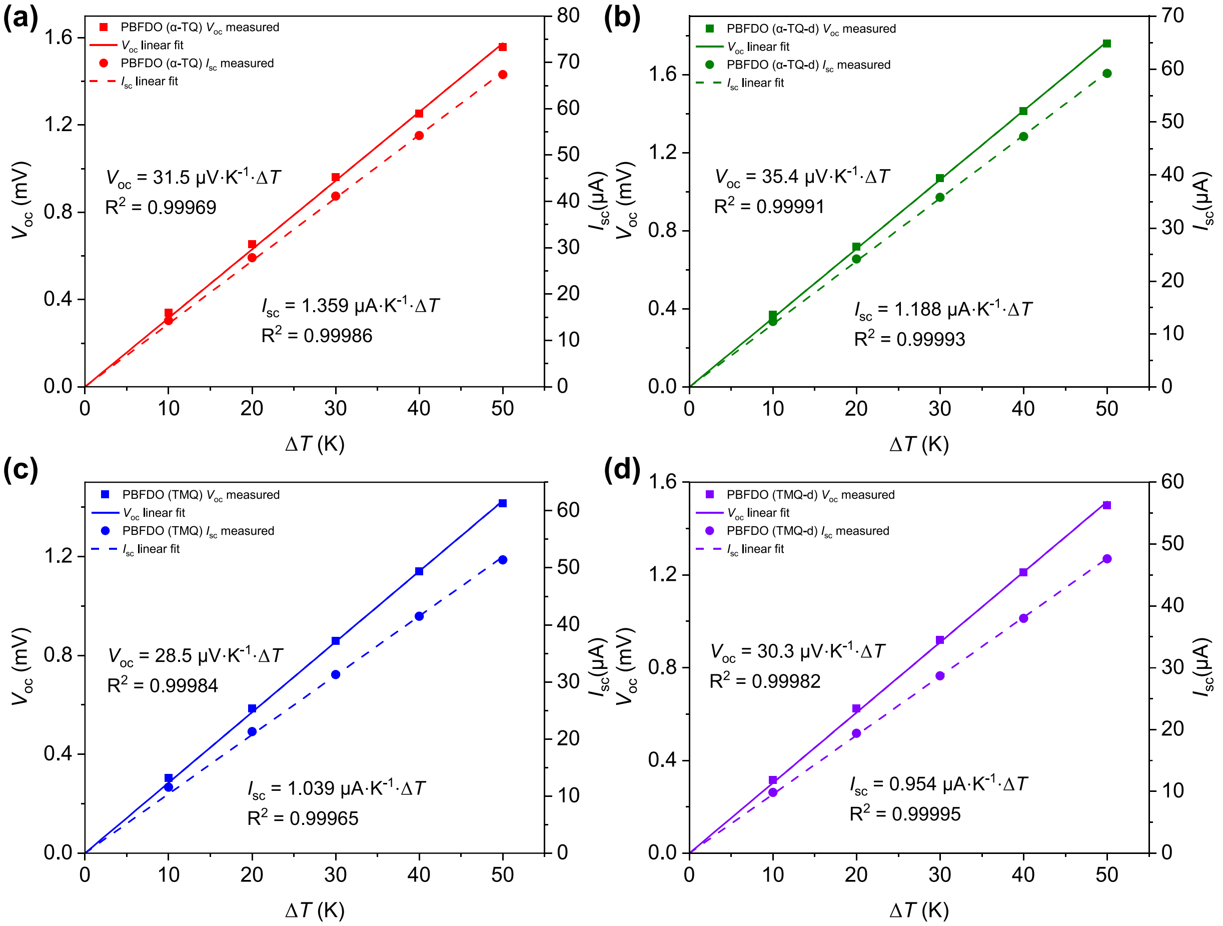

Figure S20. Open circuit voltage and short circuit current of a planar thermocouple integrating PEDOT:PSS (5 vol% EG) as the p-leg and a) α-TQ-synthesized PBFDO (before dialysis), b) α-TQ-synthesized PBFDO (after dialysis), c) TMQ-synthesized PBFDO (before dialysis), and d) TMQ-synthesized PBFDO (after dialysis) as the n-leg. All measurements were performed using gold contacts, and data are shown as a function of the applied temperature gradient.

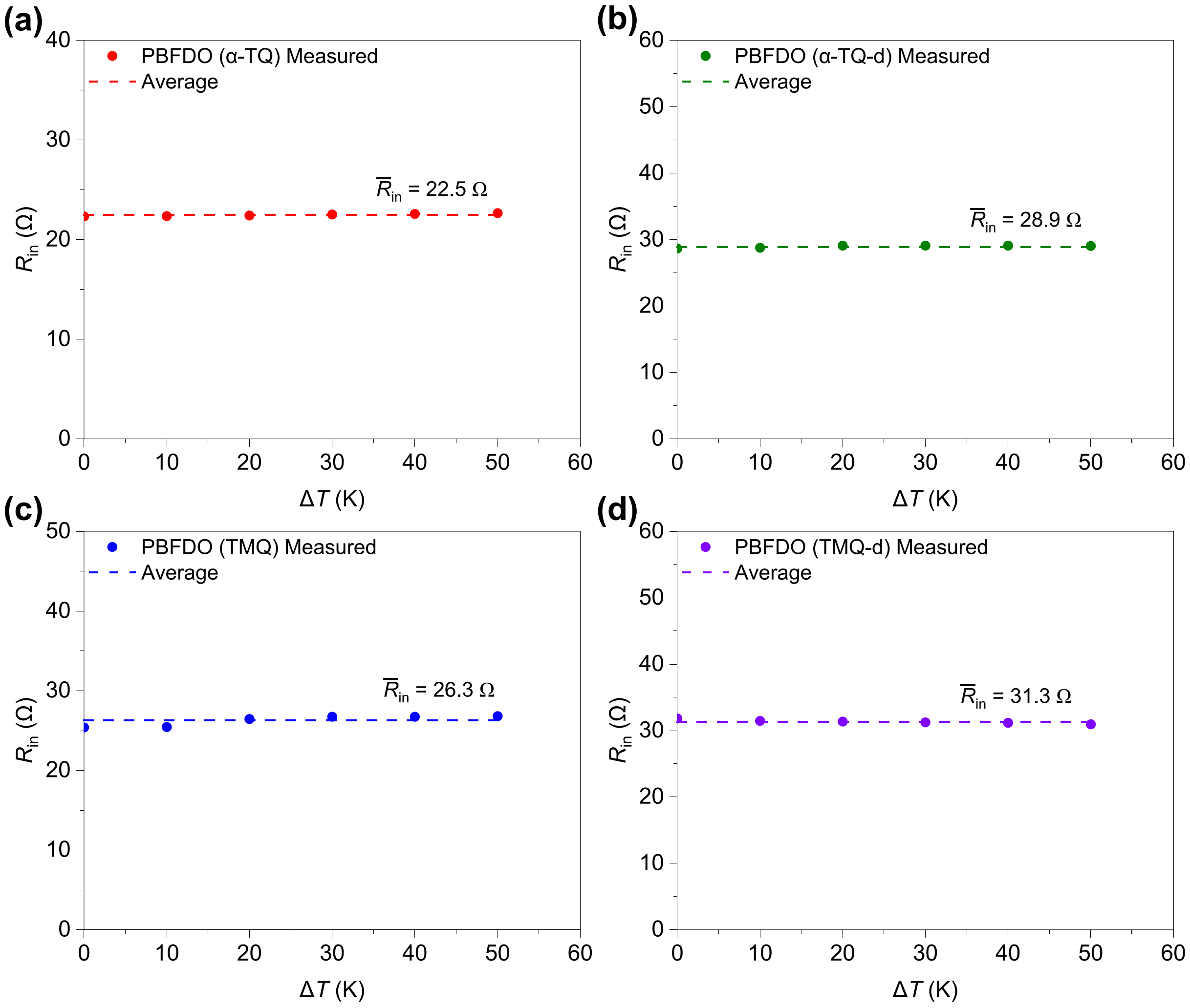

Figure S21. Internal resistance of a planar thermocouple integrating PEDOT:PSS (5 vol% EG) as the p-leg and a) α-TQ-synthesized PBFDO (before dialysis), b) α-TQ-synthesized PBFDO (after dialysis), c) TMQ-synthesized PBFDO (before dialysis), and d) TMQ-synthesized PBFDO (after dialysis) as the n-leg. All measurements were performed using gold contacts. The results show stable internal resistance across all thermocouples during measurements.

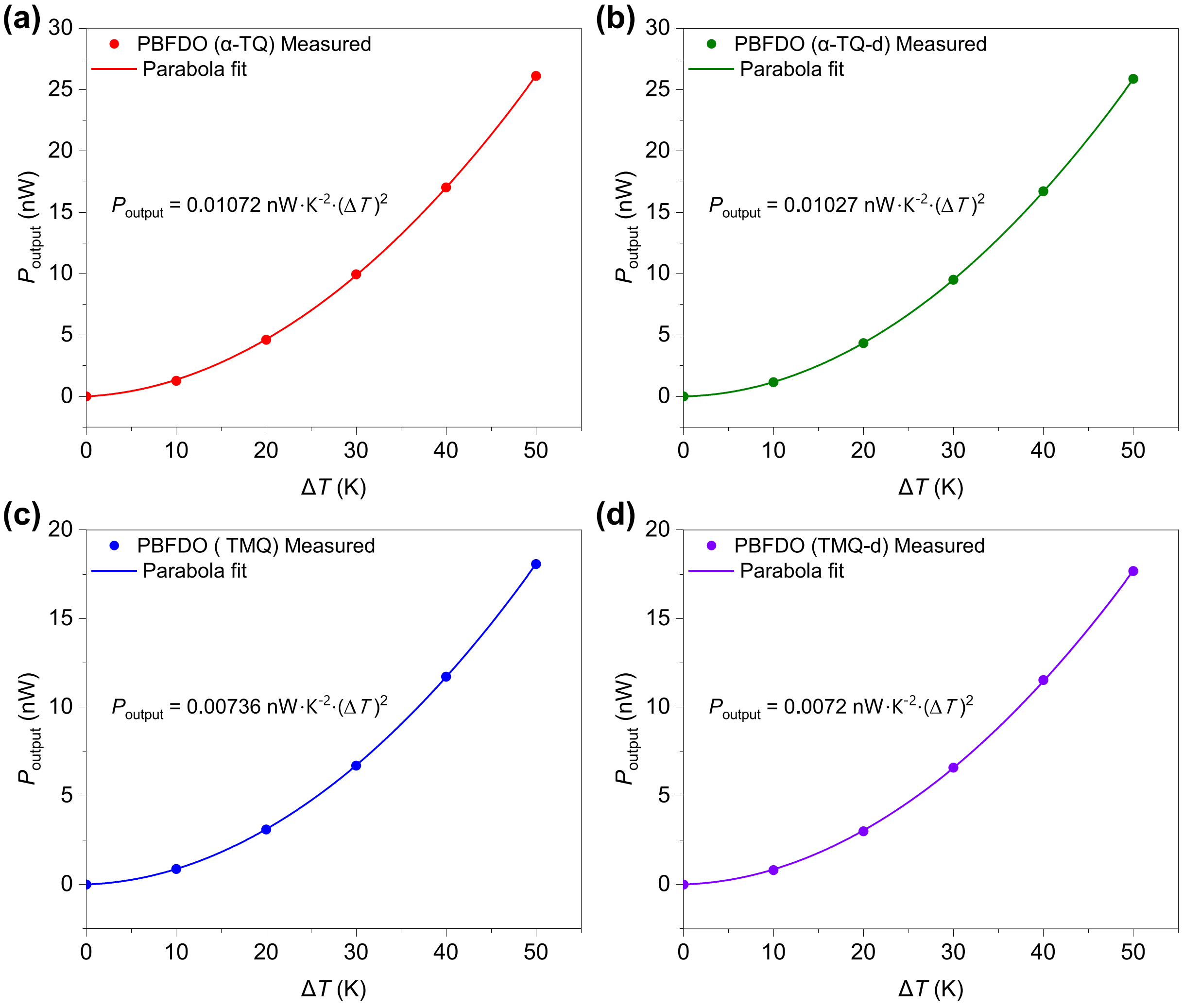

Figure S22. Output power of a planar thermocouple integrating PEDOT:PSS (5 vol% EG) as the p-leg and a) α-TQ-synthesized PBFDO (before dialysis), b) α-TQ-synthesized PBFDO (after dialysis), c) TMQ-synthesized PBFDO (before dialysis), and d) TMQ-synthesized PBFDO (after dialysis) as the n-leg. All measurements were performed using gold contacts, and data are shown as a function of the applied temperature gradient.

Table S1. Summary of the power factor of α-TQ-synthesized PBFDO (before and after dialysis) and TMQ-synthesized PBFDO (before and after dialysis).

Polymer	PBFDO (α-TQ)	PBFDO (α-TQ-d)	PBFDO (TMQ)	PBFDO (TMQ-d)
Power factor [$\mu W m^{-1} K^{-2}$]	100.4 ± 8.8	111.2 ± 6.9	16.4 ± 4.9	80.4 ± 6.2

Table S2. Summary of the *PF* of representative solution-processed n-type materials.

Material	*PF* [μWm^{-1} K^{-2}]	Year	Reference
α-TQ-synthesized PBFDO (before dialysis)	100.4	2025	This work
α-TQ-synthesized PBFDO (after dialysis)	111.2	2025	This work
f-BTI2g-SVSCN	57.9	2024	[25]
f-BSeI2g-SVSCN	114.1	2024	[25]
pDFSe	133.1	2024	[26]
ThDPP-CNBTz	126.8	2024	[27]
P(DPP-CNPz)	41.4	2024	[28]
P(DPP-DCNPz)	30.4	2024	[28]
TBDOPV-T-518	200	2023	[29]
PCNI2-V	0.11	2023	[6]
PCNI2-T	5.29	2023	[6]
PCNI2-BTI	110.3	2023	[6]
n-PT3	110.0	2023	[30]
n-PT4	157.3	2023	[30]
f-BSeI2TEG-T	8.8	2023	[31]
f-BSeI2TEG-FT	70.1	2023	[31]
PBFDO	90	2022	[1]
p(g7NC4N)	10.4	2022	[32]
PCNI-BTI	10.0	2021	[33]
PCNDTI-BTI	0.33	2021	[33]
BBL	11	2021	[2]
UFBDPPV	80	2020	[34]
LPPV-1	33.9	2020	[34]
P(PzDPP-CT2)	57.3	2019	[35]
PDPF	4.65	2018	[36]
PNDTI-BBT-DT	0.6	2017	[37]
PNDTI-BBT-DP	14	2017	[37]
FBDPPV	28	2015	[38]
P(NDIOD-T2)	0.6	2014	[39]

Table S3. Thickness measurements of p- and n-legs of the thermocouple.

Material	p-leg thickness [μm]	n-leg thickness [μm]
PBFDO (α-TQ)	3.8	2.4
PBFDO (α-TQ-d)	3.6	2.3
PBFDO (TMQ)	3.7	7.0
PBFDO (TMQ-d)	3.6	1.9

Table S4. The contribution of the p-/n-legs, electrodes and the contact resistance between them to the internal resistance.

Resistance	Device 1	Device 2	Device 3	Device 4	Average
p-leg contribution [%]	19.5	20.1	20.8	21.0	20.4
n-leg contribution [%]	30.4	28.4	27.9	31.7	29.6
eletrodes contribution [%]	11.3	11.1	11.8	11.6	11.5
contact resistance contribution [%]	38.8	40.4	39.4	35.7	38.6

Table S5. Elemental analysis of TMQ-synthesized PBFDO (after dialysis) and α-TQ-synthesized PBFDO (after dialysis).

Product	Experimental [%]		
	C	H	O
PBFDO (TMQ-d)	59.35	2.04	38.42
PBFDO (α-TQ-d)	60.57	2.37	36.63

References

[1] H. Tang, Y. Liang, C. Liu, Z. Hu, Y. Deng, H. Guo, Z. Yu, A. Song, H. Zhao, D. Zhao, Y. Zhang, X. Guo, J. Pei, Y. Ma, Y. Cao, F. Huang, *Nature* **2022**, *611*, 271.

[2] C.-Y. Yang, M.-A. Stoeckel, T.-P. Ruoko, H.-Y. Wu, X. Liu, N. B. Kolhe, Z. Wu, Y. Puttisong, C. Musumeci, M. Massetti, H. Sun, K. Xu, D. Tu, W. M. Chen, H. Y. Woo, M. Fahlman, S. A. Jenekhe, M. Berggren, S. Fabiano, *Nat. Commun.* **2021**, *12*, 2354.

[3] J. Han, Y. Jiang, E. Tiernan, C. Ganley, Y. Song, T. Lee, A. Chiu, P. McGuiggan, N. Adams, P. Clancy, T. P. Russell, P. E. Hopkins, S. M. Thon, J. D. Tovar, H. E. Katz, *Angew. Chem. Int. Ed.* **2023**, *135*, e202219313.

[4] Z. Wu, C. Sun, S. Dong, X.-F. Jiang, S. Wu, H. Wu, H.-L. Yip, F. Huang, Y. Cao, *J. Am. Chem. Soc.* **2016**, *138*, 2004.

[5] D. Jeong, I. Jo, S. Lee, J. H. Kim, Y. Kim, D. Kim, J. R. Reynolds, M. Yoon, B. J. Kim, *Adv. Funct. Mater.* **2022**, *32*, 2111950.

[6] K. Feng, W. Yang, S. Y. Jeong, S. Ma, Y. Li, J. Wang, Y. Wang, H. Y. Woo, P. K. L. Chan, G. Wang, X. Guo, M. Zhu, *Adv. Mater.* **2023**, *35*, 2210847.

[7] H. Tang, Z. Liu, Y. Tang, Z. Du, Y. Liang, Z. Hu, K. Zhang, F. Huang, Y. Cao, *Giant* **2021**, *6*, 100053.

[8] Q. Li, J.-D. Huang, T. Liu, T. P. A. Van Der Pol, Q. Zhang, S. Y. Jeong, M.-A. Stoeckel, H.-Y. Wu, S. Zhang, X. Liu, H. Y. Woo, M. Fahlman, C.-Y. Yang, S. Fabiano, *J. Am. Chem. Soc.* **2024**, *146*, 15860.

[9] M. Craighero, Q. Li, Z. Zeng, C. Choi, Y. Kim, H. Yoon, T. Liu, P. Sowinski, S. Haraguchi, B. Hwang, B. Mihiretie, S. Fabiano, C. Müller, *Adv. Sci.* **2024**, *11*, 2406770.

[10] R. Sarabia-Riquelme, L. E. Noble, P. Alarcon Espejo, Z. Ke, K. R. Graham, J. Mei, A. F. Paterson, M. C. Weisenberger, *Adv. Funct. Mater.* **2023**, *34*, 2311379.

[11] A. Lund, N. M. van der Velden, N.-K. Persson, M. M. Hamedi, C. Müller, *Mater. Sci. Engin. R: Reports* **2018**, *126*, 1.

[12] Y. Kim, A. Lund, H. Noh, A. I. Hofmann, M. Craighero, S. Darabi, S. Zokaei, J. I. Park, M. Yoon, C. Müller, *Macromol. Mater. Eng.* **2020**, *305*, 1900749.

[13] N. Kim, S. Lienemann, I. Petsagkourakis, D. Alemu Mengistie, S. Kee, T. Ederth, V. Gueskine, P. Leclère, R. Lazzaroni, X. Crispin, K. Tybrandt, *Nat. Commun.* **2020**, *11*, 1424.

[14] R. Sarabia-Riquelme, R. Andrews, J. E. Anthony, M. C. Weisenberger, *J. Mater. Chem. C* **2020**, *8*, 11618.

[15] R. Sarabia-Riquelme, M. Shahi, J. W. Brill, M. C. Weisenberger, *ACS Appl. Polym. Mater.* **2019**, *1*, 2157.

[16] J. Zhang, S. Seyedin, S. Qin, P. A. Lynch, Z. Wang, W. Yang, X. Wang, J. M. Razal, *J. Mater. Chem. A* **2019**, *7*, 6401.

[17] Y. Wang, C. Zhu, R. Pfattner, H. Yan, L. Jin, S. Chen, F. Molina-Lopez, F. Lissel, J. Liu, N. I. Rabiah, Z. Chen, J. W. Chung, C. Linder, M. F. Toney, B. Murmann, Z. Bao, *Sci. Adv.* **2017**, *3*, e1602076.

[18] S. Seyedin, J. M. Razal, P. C. Innis, A. Jeiranikhameneh, S. Beirne, G. G. Wallace, *ACS Appl. Mater. Interfaces* **2015**, *7*, 21150.

[19] J. Zhou, E. Q. Li, R. Li, X. Xu, I. A. Ventura, A. Moussawi, D. H. Anjum, M. N. Hedhili, D.-M. Smilgies, G. Lubineau, S. T. Thoroddsen, *J. Mater. Chem. C* **2015**, *3*, 2528.

[20] X. Wang, M. Ge, G. Feng, *Fibers Polym.* **2015**, *16*, 2578.

[21] M. Z. Seyedin, J. M. Razal, P. C. Innis, G. G. Wallace, *Adv. Funct. Mater.* **2014**, *24*, 2957.

[22] R. Jalili, J. M. Razal, P. C. Innis, G. G. Wallace, *Adv. Funct. Mater.* **2011**, *21*, 3363.

[23] H. Okuzaki, Y. Harashina, H. Yan, *Eur. Polym. J.* **2009**, *45*, 256.

[24] H. Okuzaki, M. Ishihara, *Macromol. Rapid Commun.* **2003**, *24*, 261.

[25] S. Gámez-Valenzuela, J. Li, S. Ma, S. Y. Jeong, H. Y. Woo, K. Feng, X. Guo, *Angew. Chem. Int. Ed.* **2024**, *63*, e202408537.

[26] T. Shen, D. Liu, J. Zhang, Z. Wei, Y. Wang, *Angew. Chem. Int. Ed.* **2024**, *63*, e202408537.

[27] Y. Gao, Y. Ke, T. Wang, Y. Shi, C. Wang, S. Ding, Y. Wang, Y. Deng, W. Hu, Y. Geng, *Angew. Chem. Int. Ed.* **2024**, *63*, e202402642.

[28] L. Tu, J. Wang, Z. Wu, J. Li, W. Yang, B. Liu, S. Wu, X. Xia, Y. Wang, H. Y. Woo, Y. Shi, *Angew. Chem. Int. Ed.* **2024**, *136*, e202319658.

[29] Z.-D. Yu, Y. Lu, Z.-Y. Wang, H.-I. Un, S. J. Zelewski, Y. Cui, H.-Y. You, Y. Liu, K.-F. Xie, Z.-F. Yao, Y.-C. He, J.-Y. Wang, W.-B. Hu, H. Sirringhaus, J. Pei, *Sci. Adv.* **2023**, *9*, eadf3495.

[30] S. Deng, Y. Kuang, L. Liu, X. Liu, J. Liu, J. Li, B. Meng, C. Di, J. Hu, J. Liu, *Adv. Mater.* **2024**, *36*, 2309679.

[31] J. Li, M. Liu, K. Yang, Y. Wang, J. Wang, Z. Chen, K. Feng, D. Wang, J. Zhang, Y. Li, H. Guo, Z. Wei, X. Guo, *Adv. Funct. Mater.* **2023**, *33*, 2213911.

[32] A. Marks, X. Chen, R. Wu, R. B. Rashid, W. Jin, B. D. Paulsen, M. Moser, X. Ji, S. Griggs, D. Meli, X. Wu, H. Bristow, J. Strzalka, N. Gasparini, G. Costantini, S. Fabiano, J. Rivnay, I. McCulloch, *J. Am. Chem. Soc.* **2022**, *144*, 4642.

[33] K. Feng, H. Guo, J. Wang, Y. Shi, Z. Wu, M. Su, X. Zhang, J. H. Son, H. Y. Woo, X. Guo, *J. Am. Chem. Soc.* **2021**, *143*, 1539.

[34] Y. Lu, Z.-D. Yu, Y. Liu, Y.-F. Ding, C.-Y. Yang, Z.-F. Yao, Z.-Y. Wang, H.-Y. You, X.-F. Cheng, B. Tang, J.-Y. Wang, J. Pei, *J. Am. Chem. Soc.* **2020**, *142*, 15340.

[35] X. Yan, M. Xiong, J.-T. Li, S. Zhang, Z. Ahmad, Y. Lu, Z.-Y. Wang, Z.-F. Yao, J.-Y. Wang, X. Gu, T. Lei, *J. Am. Chem. Soc.* **2019**, *141*, 20215.

[36] C. Yang, W. Jin, J. Wang, Y. Ding, S. Nong, K. Shi, Y. Lu, Y. Dai, F. Zhuang, T. Lei, C. Di, D. Zhu, J. Wang, J. Pei, *Adv. Mater.* **2018**, *30*, 1802850.

[37] Y. Wang, M. Nakano, T. Michinobu, Y. Kiyota, T. Mori, K. Takimiya, *Macromolecules* **2017**, *50*, 857.

[38] K. Shi, F. Zhang, C.-A. Di, T.-W. Yan, Y. Zou, X. Zhou, D. Zhu, J.-Y. Wang, J. Pei, *J. Am. Chem. Soc.* **2015**, *137*, 6979.

[39] R. A. Schlitz, F. G. Brunetti, A. M. Glaudell, P. L. Miller, M. A. Brady, C. J. Takacs, C. J. Hawker, M. L. Chabinyc, *Adv. Mater.* **2014**, *26*, 2825.
